# GLP-1 RAs and Cardiovascular and Kidney Outcomes by Body Mass Index in Type 2 Diabetes

**DOI:** 10.1001/jamanetworkopen.2025.30952

**Published:** 2025-09-08

**Authors:** Tien-Hsing Chen, En-Hao Hu, Dong-Yi Chen, Yuan Lin, Tien-Shin Chou, Ming-Shyan Lin, Ning-I. Yang, Chao-Yung Wang, Ming-Jui Hung, Ming-Lung Tsai

**Affiliations:** 1 Division of Cardiology, Department of Internal Medicine, Keelung Chang Gung Memorial Hospital, Keelung, Taiwan; 2 Department of Internal Medicine, Keelung Chang Gung Memorial Hospital, Keelung, Taiwan; 3 Division of Cardiology, Department of Internal Medicine, Linkou Chang Gung Memorial Hospital, Taoyuan, Taiwan; 4 Department of Emergency Medicine, Keelung Chang Gung Memorial Hospital, Keelung, Taiwan; 5 Division of Gastroenterology, Department of Internal Medicine, Keelung Chang Gung Memorial Hospital, Keelung, Taiwan; 6 Division of Cardiology, Department of Internal Medicine, Chiayi Chang Gung Memorial Hospital, Chiayi, Taiwan; 7 Division of Cardiology, Department of Internal Medicine, New Taipei Municipal TuCheng Hospital, New Taipei, Taiwan; 8 College of Medicine, Chang Gung University, Taoyuan, Taiwan; 9 College of Management, Chang Gung University, Taoyuan, Taiwan; 10 School of Traditional Chinese Medicine, Chang Gung University, Taoyuan, Taiwan

## Abstract

**Importance:**

The cardiovascular benefits of glucagon-like peptide-1 receptor agonists (GLP-1 RAs) may vary by body mass index (BMI), but evidence on BMI-specific outcomes remains limited.

**Objective:**

To investigate the associations of GLP-1 RA use with cardiovascular and kidney outcomes across BMI categories in patients with type 2 diabetes.

**Design, Setting, and Participants:**

This retrospective cohort study used the Chang Gung Research Database, a clinical dataset covering multiple hospitals in Taiwan. Patients with type 2 diabetes who received GLP-1 RAs or dipeptidyl peptidase-4 (DPP-4) inhibitors between 2011 and 2022 were identified. DPP-4 inhibitors were selected as the comparator due to their widespread use as a second-line oral hypoglycemic agent and their relatively neutral cardiovascular and kidney effects reported in previous studies. Propensity score matching was applied separately within BMI categories less than 25 and 25 or greater using a comprehensive set of demographic, clinical, and biochemical variables to balance baseline characteristics between treatment groups. The analysis was conducted from December 15, 2023, to July 5, 2024.

**Exposures:**

Initiation of GLP-1 RAs compared with DPP-4 inhibitors.

**Main Outcomes and Measures:**

Primary outcomes included major adverse cardiovascular events (MACE; defined as cardiovascular death, myocardial infarction, ischemic stroke, or hospitalization for heart failure) and composite kidney outcomes (defined as estimated glomerular filtration rate decline ≥50% or progression to dialysis).

**Results:**

Among 97 156 patients with diabetes identified, a total of 7200 matched patients (mean [SD] age, 57.4 [14.2] years; 7473 [51.9%] female) were included (1841 pairs with BMI <25 and 5359 pairs with BMI ≥25). Among patients with BMI 25 or greater, GLP-1 RAs were associated with lower risks of cardiovascular death (hazard ratio [HR], 0.62; 95% CI, 0.46-0.83) and hospitalization for heart failure (subdistribution HR, 0.77; 95% CI, 0.62-0.94). Kidney outcomes were consistent across BMI strata. Restricted cubic spline analysis revealed increasing cardiovascular benefit associated with GLP-1 RAs among patients with higher BMI.

**Conclusions and Relevance:**

In this cohort study of patients with type 2 diabetes, GLP-1 RAs use was associated with BMI-dependent cardiovascular benefits and consistent kidney protection, suggesting the importance of BMI stratification in guiding treatment decisions.

## Key Points

**Question:**

Are glucagon-like peptide-1 receptor agonists (GLP-1 RAs) associated with cardiovascular and kidney outcomes in patients with type 2 diabetes, and do these associations differ by body mass index (BMI)?

**Findings:**

In this cohort study of 97 156 adults with type 2 diabetes, use of GLP-1 RAs was associated with significantly lower risks of major adverse cardiovascular events, cardiovascular death, and hospitalization for heart failure among patients with BMI of 25 or greater. Kidney benefits associated with GLP-1 RAs were consistent across BMI categories.

**Meaning:**

These findings suggest that cardiovascular benefits of GLP-1 RAs may be modified by BMI, while kidney benefits were consistent regardless of BMI, supporting individualized treatment decisions in type 2 diabetes care.

## Teaser

This cohort study examines associations of glucagon-like peptide-1 receptor agonist (GLP-1 RA) use with cardiovascular and kidney outcomes across body mass index (BMI) categories in patients with type 2 diabetes.

## Introduction

Type 2 diabetes is a major global public health issue, with cardiovascular (CV) and kidney complications contributing to its disease burden.^1,2^ Glucagon-like peptide-1 receptor agonists (GLP-1 RAs) have emerged as an important therapeutic class, offering both glycemic control and potential cardioprotective and nephroprotective benefits.^3,4,5^ However, GLP-1 RAs are not a pharmacologically homogenous class, and their effects on specific CV outcomes appear to vary among agents and patient populations. For instance, semaglutide induces greater weight reduction than liraglutide, which may lead to distinct cardiorenal outcomes, especially in patients with varying body mass index (BMI; calculated as weight in kilograms divided by height in meters squared). In patients with heart failure (HF) with reduced ejection fraction, the LIVE trial^6^ showed no improvement in left ventricular ejection fraction with liraglutide and even reported increased cardiac adverse events. Similarly, the FIGHT trial^7,8^ failed to demonstrate reductions in hospitalization for HF (HHF) or improvement in natriuretic peptides.

In contrast, evidence suggests that GLP-1 RAs may provide CV benefits in patients with obesity. The STEP-HFpEF trial^9^ demonstrated that semaglutide improved heart failure symptoms in patients with obesity without diabetes and with preserved ejection fraction. Furthermore, the SELECT trial^10^ showed that semaglutide reduced major adverse CV events (MACE) in individuals with overweight or obesity without diabetes.^10^ These findings suggest a potential interaction between BMI and the cardioprotective effects of GLP-1 RAs. However, few studies have directly examined this interaction in patients with type 2 diabetes.

We hypothesized that BMI may modify the CV and kidney outcomes associated with GLP-1 RAs in patients with type 2 diabetes. To evaluate this hypothesis, we conducted a large-scale retrospective cohort study comparing GLP-1 RAs with dipeptidyl peptidase-4 (DPP-4) inhibitors, a commonly used second-line therapy in Taiwan with a well-established neutral CV and kidney profile.^11,12^ We analyzed data from a multi-institutional health care system, stratifying by BMI to evaluate whether treatment outcomes differed across weight categories.

## Method

This cohort study was approved by the ethical review board of Chang Gung Medical Foundation with a waiver of informed consent because data were deidentified. This cohort study was conducted in accordance with the Declaration of Helsinki and followed the Strengthening the Reporting of Observational Studies in Epidemiology (STROBE) reporting guideline.

### Patients and Study Design

We conducted a retrospective cohort study using a new-user, active comparator design.^13^ Participants were patients with type 2 diabetes who initiated GLP-1 RAs or DPP-4 inhibitors in Chang-Gung Memorial Hospitals from December 15, 2011, to December 31, 2022. As the analysis was based on a clinical population with fixed inclusion criteria, no formal sample size calculation was performed. Key exclusion criteria included age younger than 20 years, type 1 diabetes, HF with reduced ejection fraction (<40%), bariatric surgery, prior use of sodium-glucose cotransporter 2 inhibitors, missing baseline hemoglobin A_1c_ or BMI, follow-up less than 90 days, and treatment switching within 90 days (eMethods in Supplement 1). Patients were stratified by BMI (≥25 vs <25) and treatment group (GLP-1 RAs vs DPP-4 inhibitors).^14,15^

### Data Source

We used the Chang Gung Research Database, a multi-institutional electronic medical record database managed by the Chang Gung Medical Foundation health care system in Taiwan. All data were anonymized and deidentified. Details regarding the Chang Gung Research Database have been published elsewhere.^16,17^ Detailed information is provided in eMethods in Supplement 1.

### Covariates and Baseline Characteristics

Baseline characteristics included demographics, comorbidities, laboratory values, and medications, all selected based on clinical relevance and previous literature on cardiorenal risk factors.^18^ These covariates were used for propensity score estimation and adjusted for in all outcome models. Details regarding covariate definitions and diagnostic coding are provided in the eMethods and eTable 1 in Supplement 1.

### Outcomes Definition

Primary outcomes were MACE, including CV death, myocardial infarction, ischemic stroke, and HHF, and composite kidney outcomes, defined as at least 50% decline in estimated glomerular filtration rate (eGFR) or progression to dialysis. Secondary outcomes included all-cause mortality, infection-related admission, composite lower-limb events, hypoglycemia, diabetic ketoacidosis (DKA) and hyperosmolar hyperglycemic state (HHS), and pancreatitis. Detailed information is provided in the eMethods in Supplement 1. Mortality data were linked to the national death registry. Patients were followed-up from the date of initial dispensation (the index date) until the day of outcome occurrence, drug switch (eg, DPP4 inhibitor to GLP-1 RA), death, or the end of the database (December 31, 2022), whichever came first.

### Statistical Analysis

We conducted 1:1 propensity score matching to reduce confounding between treatment groups. Matching was based on clinically relevant variables using a nearest-neighbor algorithm with a caliper of 0.2.^19^ Matching was conducted in a random order without replacement to ensure unbiased pair selection and performed separately for the nonobese and obese groups. Covariate balance was assessed using standardized mean differences.

Outcomes were analyzed in the matched cohort using Cox proportional hazards models for mortality and Fine-Gray subdistribution models for nonfatal events, accounting for the competing risk of death.^20^ BMI was also modeled as a continuous variable using restricted cubic splines to evaluate interaction effects. A 2-sided *P* < .05 was considered statistically significant.

Additional details on the propensity score estimation, missing data imputation, and spline model specifications are provided in the eMethods in Supplement 1. The restricted cubic spline analysis was carried out using R version 4.3.2 (R Project for Statistical Computing) with the rms and interactionRCS packages. The remaining analyses (including matching) were conducted using SAS software version 9.4 (SAS Institute). Analyses were conducted from December 15, 2023, to July 5, 2024.

Sensitivity analyses included comparisons of individual GLP-1 RAs (liraglutide and dulaglutide), stratified analyses by prior sodium-glucose cotransporter 2 inhibitor use and concomitant insulin use, exclusion of thiazolidinediones or pancreatitis patients, inverse probability of treatment weighting, and multiple imputation for missing data. Full details are provided in the eMethods in Supplement 1.

## Results

### Patient Selection and Demographic Data

**Figure 1.**
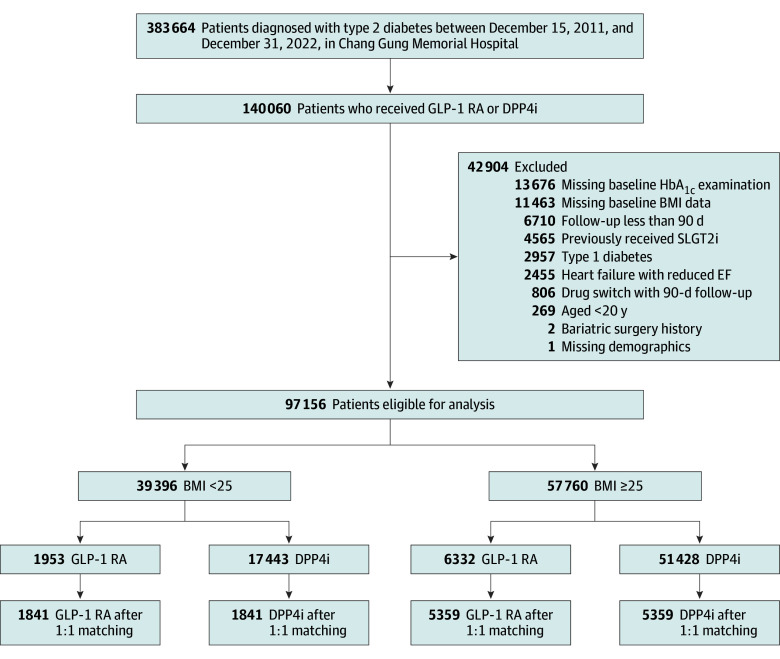
Flow of Patient Inclusion and Exclusion BMI indicates body mass index (calculated as weight in kilograms divided by height in meters squared); DPP4i, DPP-4 inhibitors; EF, ejection fraction; GLP-1 RA, glucagon-like peptide-1 receptor agonist; HbA_1c_, glycated hemoglobin A_1c_; SLGT2i, sodium–glucose cotransporter 2 inhibitor.

We identified 97 156 patients with type 2 diabetes who received their first prescription for GLP-1RAs or DPP-4 inhibitors between December 15, 2011, and December 31, 2022 (Figure 1). Among 8285 patients using GLP-1 RAs, dulaglutide (3433 patients [41.6%]) and liraglutide (2734 patients [33.1%]) were the most prescribed, followed by semaglutide (1239 patients [15.0%]), Soliqua (a fixed combination of lixisenatide and insulin glargine; 694 patients [8.4%]), and exenatide (185 patients [2.2%]). Before propensity score matching, GLP-1 RAs users were generally younger and had longer diabetes duration, higher rates of diabetic complications, and more frequent use of thiazolidinedione and insulin (Table 1). After matching, a total of 7200 matched patients (mean [SD] age, 57.4 [14.2] years; 7473 [51.9%] female) were included (1841 pairs with BMI <25 and 5359 pairs with BMI ≥25). Baseline characteristics were well balanced across groups in each BMI category (eTable 2 in Supplement 1).

**Table 1.** Baseline Characteristics of Patients With Type 2 Diabetes Treated With GLP-1 RAs vs DPP4i Stratified by BMI

Variable	BMI <25				BMI ≥25				
	Available No.	Patients, No. (%)		STD	Available No.	Patients, No. (%)		STD
		GLP-1 RAs (n = 1953)	DPP4i (n = 37 443)			GLP-1 RAs (n = 6332)	DPP4i (n = 51 428)	
Age, mean (SD), y	39 396	61.0 (14.2)	66.1 (12.4)	−0.38	57 760	55.6 (14.0)	62.2 (12.9)	−0.49
Sex								
Female	39 396	1062 (54.4)	17 422 (46.5)	0.16	57 760	3252 (51.4)	22 503 (43.8)	0.15
Male	39 396	891 (45.6)	20 021 (53.5)	−0.16	57 760	3080 (48.6)	28 925 (56.2)	−0.15
Smoking	39 396	307 (15.7)	6688 (17.9)	−0.06	57 760	1054 (16.6)	9071 (17.6)	−0.03
Alcohol consumption	39 396	149 (7.6)	3832 (10.2)	−0.09	57 760	504 (8.0)	5422 (10.5)	−0.09
BMI, mean (SD)	39 396	22.7 (1.9)	22.4(2.0)	0.15	57 760	31.1 (5.0)	29.1 (3.6)	0.46
Established ASCVD^a^	39 396	651 (33.3)	11 475 (30.6)	0.06	57 760	1835 (29.0)	16 604 (32.3)	−0.07
Multiple risk factors for ASCVD^b^	39 396	506 (25.9)	10 937 (29.2)	−0.07	57 760	1317 (20.8)	13 435 (26.1)	−0.13
Severity of diabetes								
Insulin use	39 396	613 (31.4)	5644 (15.1)	0.39	57 760	1583 (25.0)	5305 (10.3)	0.39
Diabetes duration, y								
Mean (SD)	39 396	8.9 (6.7)	5.0(5.4)	0.64	57 760	7.6 (6.2)	4.2 (4.9)	0.60
Median (IQR)	39 396	9.0 (2.1-15.2)	3.2 (0.1-9.2)	NA	57 760	7.0 (1.4-12.6)	2.5 (0.1-7.4)	NA
Glycated hemoglobin, mean (SD), %	39 396	8.8 (2.1)	8.4 (2.1)	0.18	57 760	8.7 (2.0)	8.3 (1.8)	0.19
Diabetic nephropathy	39 396	704 (36.0)	6489 (17.3)	0.43	57 760	2103 (33.2)	8301 (16.1)	0.40
Diabetic retinopathy	39 396	435 (22.3)	3736 (10.0)	0.34	57 760	1094 (17.3)	3776 (7.3)	0.31
Diabetic neuropathy	39 396	1012 (51.8)	9470 (25.3)	0.57	57 760	2871 (45.3)	11 181 (21.7)	0.52
Diabetic foot	39 396	103 (5.3)	1036 (2.8)	0.13	57 760	235 (3.7)	889 (1.7)	0.12
Outpatient visits for diabetes, No.								
Mean (SD)	39 396	6.1 (5.0)	3.7 (4.1)	0.52	57 760	5.9 (4.8)	3.6(3.8)	0.55
Median (IQR)	39 396	5.0 (3.0-9.0)	3.0 (0.0-5.0)	NA	57 760	5.0 (3.0-8.0)	3.0 (0.0-5.0)	NA
LVEF								
Mean (SD), %	12 704	67.3 (9.9)	67.8 (9.8)	−0.05	19 142	67.8 (8.8)	68.0 (9.2)	−0.02
<50%	12 704	36 (6.2)	723 (6.0)	0.01	19 142	65 (3.4)	720 (4.2)	0.04
≥50%	12 704	546 (93.8)	11 399 (94.0)		19 142	1821 (96.6)	16 536 (95.8)	
HHF in the previous 1 y	39 396	56 (2.9)	966 (2.6)	0.02	57 760	96 (1.5)	931 (1.8)	−0.02
HHF history	39 396	105 (5.4)	1555 (4.2)	0.06	57 760	269 (4.2)	1900 (3.7)	0.03
Serum creatinine, mean (SD), mg/dL	37 623	1.2 (0.9)	1.1 (0.9)	0.06	55 384	1.1 (0.7)	1.1 (0.8)	−0.02
eGFR, ml/min/1.73m^2^	37 623	83.1 (39.7)	85.8 (38.9)	−0.07	55 384	86.5 (37.2)	84.4 (35.4)	0.06
eGFR stage								
≥60 mL/min/1.73m^2^	38 398	1275 (67.4)	26 689 (73.1)	−0.13	56 038	4515 (73.6)	37 448 (75.0)	−0.03
30-59 mL/min/1.73m^2^	38 398	380 (20.1)	6515 (17.8)	0.06	56 038	1099 (17.9)	8934 (17.9)	<0.01
<30 mL/min/1.73m^2^	38 398	167 (8.8)	2597 (7.1)	0.06	56 038	393 (6.4)	2995 (6.0)	0.02
Dialysis	38 398	71 (3.8)	704 (1.9)	0.11	56 038	128 (2.1)	526 (1.1)	0.08
Baseline comorbidity								
Hypertension	39 396	1185 (60.7)	22 962 (61.3)	−0.01	57 760	4532 (71.6)	37 207 (72.3)	−0.02
Dyslipidemia	39 396	1393 (71.3)	19 760 (52.8)	0.39	57 760	4800 (75.8)	31 312 (60.9)	0.32
Coronary artery disease	39 396	463 (23.7)	6934 (18.5)	0.13	57 760	1391 (22.0)	11 532 (22.4)	−0.01
Ischemic stroke	39 396	165 (8.4)	3924 (10.5)	−0.07	57 760	346 (5.5)	4524 (8.8)	−0.13
Myocardial infarction	39 396	117 (6.0)	1514 (4.0)	0.09	57 760	271 (4.3)	2079 (4.0)	0.01
Coronary intervention	39 396	190 (9.7)	2201 (5.9)	0.14	57 760	475 (7.5)	3362 (6.5)	0.04
Peripheral artery disease	39 396	154 (7.9)	1930 (5.2)	0.11	57 760	361 (5.7)	2103 (4.1)	0.07
Critical limb ischemia	39 396	55 (2.8)	514 (1.4)	0.10	57 760	110 (1.7)	394 (0.8)	0.09
Atrial fibrillation	39 396	77 (3.9)	1751 (4.7)	−0.04	57 760	194 (3.1)	2262 (4.4)	−0.07
Gout	39 396	236 (12.1)	3764 (10.1)	0.06	57 760	1091 (17.2)	7574 (14.7)	0.07
Pancreatitis	39 396	58 (3.0)	968 (2.6)	0.02	57 760	177 (2.8)	971 (1.9)	0.06
Malignant neoplasm	39 396	230 (11.8)	5743 (15.3)	−0.10	57 760	560 (8.8)	6125 (11.9)	−0.10
Charlson Comorbidity Index score, mean (SD)	39 396	3.6 (2.5)	3.2 (2.3)	0.14	57 760	3.3 (2.3)	3.0 (2.1)	0.11
Baseline vital sign, mean (SD)								
Systolic blood pressure, mm Hg	38 809	134.8 (21.3)	135.8 (21.5)	−0.05	57 087	141.3 (20.1)	141.5 (20.3)	−0.01
Diastolic blood pressure, mm Hg	38 805	74.3 (11.8)	74.8 (11.9)	−0.04	57 082	79.4 (12.4)	78.9 (12.2)	0.04
Heart rate, beat/min	38 748	85.7 (13.6)	84.5 (14.4)	0.09	57 010	86.9 (13.6)	83.8 (14.0)	0.22
Biochemistry data, mean (SD)								
Cholesterol, mg/dL								
Low-density lipoprotein	35 366	98.1 (44.6)	102.6 (45.1)	−0.10	53 288	102.3 (49.8)	106.4 (51.2)	−0.08
High-density lipoprotein	33 535	48.3 (14.4)	46.0 (13.6)	0.16	50 296	43.3 (11.3)	43.6 (11.5)	−0.02
Total cholesterol	35 056	172.3 (43.4)	174.2 (42.6)	−0.04	52 331	174.7 (42.3)	177.5 (41.3)	−0.07
Triglyceride, mg/dL	34 892	156.4 (124.8)	148.3 (105.9)	0.07	52 370	200.8 (145.1)	179.7 (123.5)	0.16
Hemoglobin, g/dL	24 683	12.3 (2.1)	12.2 (2.2)	0.06	30 973	13.2 (2.1)	13.0 (2.3)	0.08
Uric acid, mg/dL	19 644	5.7 (1.8)	5.9 (1.9)	−0.11	31 107	6.2 (1.7)	6.3 (1.8)	−0.04
UACR, mg/g								
<30	17 109	466 (42.3)	9129 (57.0)	−0.30	26 451	1489 (40.4)	12 455 (54.7)	−0.29
30-300	17 109	382 (34.7)	4527 (28.3)	0.14	26 451	1292 (35.1)	6854 (30.1)	0.11
>300	17 109	253 (23.0)	2352 (14.7)	0.21	26 451	901 (24.5)	3460 (15.2)	0.23
Concomitant oral antiglycemic drugs								
Biguanide	39 396	1194 (61.1)	29 426 (78.6)	−0.39	57 760	4309 (68.1)	42 382 (82.4)	−0.34
Sulfonylurea	39 396	1201 (61.5)	20 537 (54.8)	0.14	57 760	3898 (61.6)	26 476 (51.5)	0.20
Thiazolidinedione	39 396	450 (23.0)	2238 (6.0)	0.50	57 760	1452 (22.9)	3634 (7.1)	0.46
α Glucosidase inhibitors	39 396	344 (17.6)	4263 (11.4)	0.18	57 760	1038 (16.4)	5099 (9.9)	0.19
Glinide	39 396	149 (7.6)	2699 (7.2)	0.02	57 760	309 (4.9)	2547 (5.0)	<0.01
Concomitant cardiovascular agents								
RAASi	39 396	867 (44.4)	16 492 (44.0)	0.01	57 760	3678 (58.1)	29 678 (57.7)	0.01
β-blocker	39 396	426 (21.8)	7618 (20.3)	0.04	57 760	1580 (25.0)	13 229 (25.7)	−0.02
Calcium channel blocker	39 396	675 (34.6)	14 474 (38.7)	−0.09	57 760	2775 (43.8)	24 805 (48.2)	−0.09
Statin	39 396	1246 (63.8)	18 363 (49.0)	0.30	57 760	4134 (65.3)	28 937 (56.3)	0.19
Fibrates	39 396	132 (6.8)	2132 (5.7)	0.04	57 760	801 (12.7)	4747 (9.2)	0.11
Aspirin	39 396	489 (25.0)	9352 (25.0)	<0.01	57 760	1498 (23.7)	14 152 (27.5)	−0.09
P2Y12 receptor blockers	39 396	235 (12.0)	3481 (9.3)	0.09	57 760	450 (7.1)	4241 (8.2)	−0.04
Oral anticoagulants	39 396	57 (2.9)	1402 (3.7)	−0.05	57 760	161 (2.5)	1877 (3.6)	−0.06
Follow-up, y								
Mean (SD)	39 396	2.5 (1.9	4.5 (3.0	−0.77	57 760	3.2 (2.5	4.9 (3.0	−0.63
Median (IQR)	39 396	2.0 (0.9-3.8)	4.1 (1.9-6.8)	NA	57 760	2.8 (1.1-4.6)	4.6 (2.3-7.4)	NA

Abbreviations: ASCVD, atherosclerotic cardiovascular disease; BMI, body mass index (calculated as weight in kilograms divided by height in meters squared); DPP4i, dipeptidyl peptidase-4 inhibitor; eGFR, estimated glomerular filtration rate; GLP-1 RA, glucagon-like peptide-1 receptor agonist; HHF, hospitalization for heart failure; LVEF, left ventricular ejection fraction; P2Y12, purinergic receptor P2Y G-protein coupled 12; RAASi, renin-angiotensin-aldosterone system inhibitor; STD, standardized difference; UACR, urine albumin to creatinine ratio.

SI conversion factors: To convert cholesterol to millimoles per liter, multiply by 0.0259; triglyceride to millimoles per liter, multiply by 0.0113; hemoglobin to grams per liter, multiply by 10; uric acid to millimoles per liter, multiply by 0.0595.

^a^ Any of coronary heart disease, coronary revascularization, ischemic stroke, intracerebral hemorrhage, carotid artery stent, myocardial infarction, peripheral artery disease, and lower-limb revascularization.

^b^ Male aged older than 55 years or female aged older than 60 years with hyperlipidemia, hypertension, and/or smoke.

### CV Outcomes

**Figure 2.**
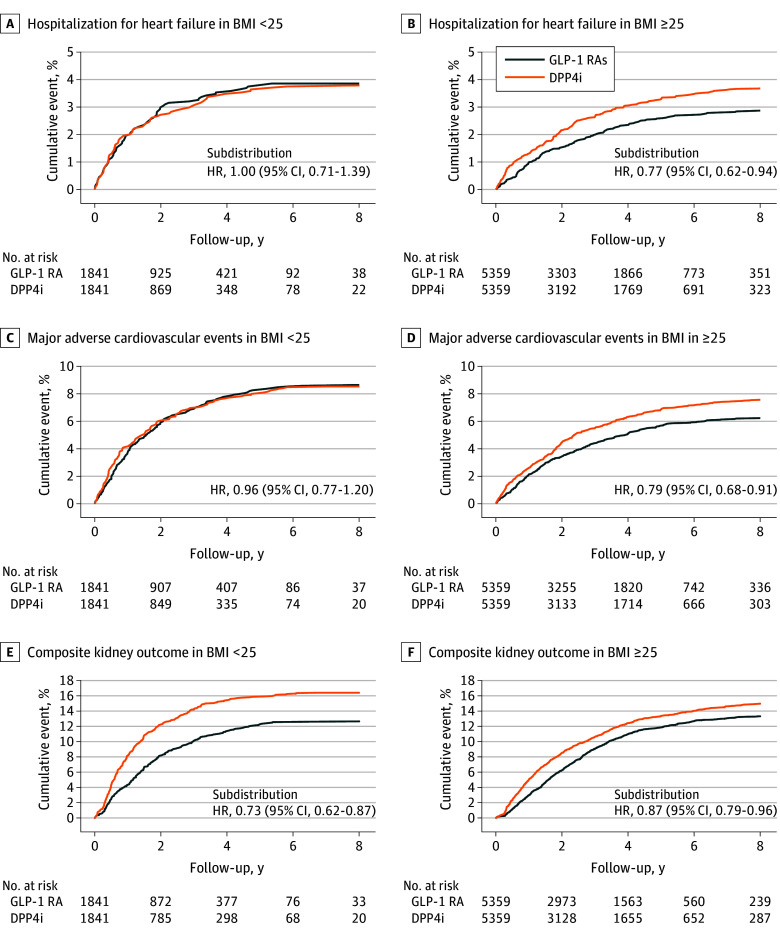
Cumulative Cardiovascular and Kidney Outcomes in the Propensity Score–Matched Cohort BMI indicates body mass index (calculated as weight in kilograms divided by height in meters squared); DPP4i, DPP-4 inhibitor; GLP-1 RA, glucagon-like peptide-1 receptor agonist.

The mean (SD) follow-up in the matched cohort was 3.1 (2.4) years. In patients with a BMI of less than 25, the risk of composite CV outcomes did not differ significantly between the GLP-1 RAs and DPP-4 inhibitors groups (8.6% vs 8.5%; hazard ratio [HR], 0.96; 95% CI, 0.77-1.20) (Table 2). Similarly, individual components of the CV outcomes, including CV death, MI, ischemic stroke, and HHF, showed no significant differences. However, in the BMI 25 or greater group, GLP-1 RA use was significantly associated with reduced risk of MACE compared with DPP-4 inhibitor use (6.3% vs 7.8%; HR, 0.79; 95% CI, 0.68-0.91), particularly noted in CV death (1.4% vs 2.2%; HR, 0.62; 95% CI, 0.46-0.83) and HHF (2.9% vs 3.8%; subdistribution HR [SHR], 0.77; 95% CI, 0.62-0.94) (Figure 2A-D).

**Table 2.** Outcomes of Patients With Diabetes Treated With GLP-1 RAs vs DPP4i in the Propensity Score–Matched Cohort Stratified by BMI

Outcome	BMI <25			BMI ≥25		
	Patients, No. (%)		GLP-1 RAs, HR/SHR (95% CI)	Patients, No. (%)		GLP-1 RAs, HR/SHR (95% CI)
	GLP-1 RAs (n = 1841)	DPP4i (n = 1841)		GLP-1 RAs (n = 5359)	DPP4i (n = 5359)	
Major adverse cardiac events						
Cardiovascular death^a^	47 (2.6)	50 (2.7)	0.89 (0.60-1.32)	74 (1.4)	116 (2.2)	0.62 (0.46-0.83)^b^
Myocardial infarction^c^^,^^d^	33 (1.8)	35 (1.9)	0.92 (0.57-1.48)	65 (1.2)	81 (1.5)	0.80 (0.58-1.11)
Ischemic stroke^c^	44 (2.4)	47 (2.6)	0.92 (0.61-1.38)	102 (1.9)	119 (2.2)	0.86 (0.66-1.12)
Hospitalization for heart failure^c^	71 (3.9)	70 (3.8)	1.00 (0.71-1.39)	157 (2.9)	204 (3.8)	0.77 (0.62-0.94)^b^
Composite MACE outcome^a^^,^^e^	159 (8.6)	157 (8.5)	0.96 (0.77-1.20)	338 (6.3)	418 (7.8)	0.79 (0.68-0.91)^b^
Kidney outcome						
eGFR decline >50%^c^	209 (11.4)	265 (14.4)	0.75 (0.62-0.90)^b^	693 (12.9)	770 (14.4)	0.88 (0.79-0.98)^b^
Progression to dialysis^c^	75 (4.1)	111 (6.0)	0.66 (0.49-0.88)^b^	215 (4.0)	268 (5.0)	0.79 (0.66-0.95)^b^
Composite renal outcomes^c^	233 (12.7)	302 (16.4)	0.73 (0.62-0.87)^b^	734 (13.7)	820 (15.3)	0.87 (0.79-0.96)^b^
Secondary outcome						
All-cause death^a^	140 (7.6)	208 (11.3)	0.64 (0.52-0.79)^b^	278 (5.2)	445 (8.3)	0.61 (0.53-0.71)^b^
Admission due to infection^c^	289 (15.7)	328 (17.8)	0.85 (0.72-0.99)^b^	757 (14.1)	877 (16.4)	0.84 (0.76-0.92)^b^
Admission due to any cause^c^	602 (32.7)	644 (35.0)	0.89 (0.79-0.99)^b^	1779 (33.2)	1842 (34.4)	0.92 (0.87-0.99)^b^
Composite MALE outcome^c^^,^^f^	76 (4.1)	70 (3.8)	1.06 (0.77-1.47)	188 (3.5)	198 (3.7)	0.94 (0.77-1.15)
Hypoglycemia^c^^,^^g^	86 (4.7)	54 (2.9)	1.57 (1.12-2.21)^b^	180 (3.4)	138 (2.6)	1.31 (1.05-1.63)^b^
DKA/HHS^c^	189 (10.3)	136 (7.4)	1.39 (1.11-1.74)^b^	455 (8.5)	354 (6.6)	1.30 (1.13-1.50)^b^
Newly diagnosed pancreatitis^c^	6 (0.3)	12 (0.7)	0.49 (0.18-1.30)	41 (0.8)	34 (0.6)	1.21 (0.77-1.91)

Abbreviations: BMI, body mass index (calculated as weight in kilograms divided by height in meters squared); DKA, diabetic ketoacidosis; DPP4i, dipeptidyl peptidase-4 inhibitor; eGFR, estimated glomerular filtration rate; GLP-1 RA, glucagon-like peptide-1 receptor agonist; HHS, hyperosmolar hyperglycemic syndrome; HR, hazard ratio; MACE, major adverse cardiovascular events; MALE, major adverse lower-limb events; SHR, subdistribution hazard ratio.

^a^ Cox proportional hazards model.

^b^ *P* < .05.

^c^ Fine and Gray subdistribution hazard model.

^d^ Hospitalization for acute myocardial infarction and troponin-I greater than 0.5 or troponin-P greater than 100.

^e^ Composite of cardiovascular death, myocardial infarction, ischemic stroke, or hospitalization for heart failure.

^f^ Anyone of newly diagnosed peripheral artery disease (including critical limb ischemia), endovascular therapy/peripheral bypass and non-traumatic major amputation.

^g^ Fasting glucose less than 54 mg/dL.

### Kidney Outcomes

GLP-1 RAs were associated with reduced risk of kidney events in both BMI groups (Table 2). In patients with BMI less than 25, fewer patients using GLP-1 RAs progressed to severe eGFR decline (11.4% vs 14.4%; SHR, 0.75; 95% CI, 0.62-0.90) or dialysis (4.1% vs 6.0%; SHR, 0.66; 95% CI, 0.49-0.88) compared with those using DPP-4 inhibitors. Similar protective associations were observed in the BMI 25 or greater group (eGFR decline >50%: 12.9% vs 14.4%; SHR, 0.88; 95% CI, 0.79-0.98; progression to dialysis: 4.0% vs 5.0%; SHR, 0.79; 95% CI, 0.66-0.95) (Figure 2E-F).

### Secondary Outcomes

GLP-1 RAs also were associated with significantly reduced risks of all-cause mortality (BMI <25: 7.6% vs 11.3%; HR 0.64; 95% CI, 0.52-0.79; BMI ≥25: 5.2% vs 8.3%; HR, 0.61; 95% CI, 0.53-0.71), admissions due to infections (BMI <25: 15.7% vs 17.8%; SHR, 0.85; 95% CI, 0.72-0.99; BMI ≥25: 14.1% vs 16.4%; SHR, 0.84; 95% CI, 0.76-0.92), and admissions due to any cause (BMI <25: 32.7% vs 35.0%; SHR, 0.89; 95% CI, 0.79-0.99; BMI ≥25: 33.2% vs 34.4%; SHR 0.92; 95% CI, 0.87-0.99) in both BMI groups compared with patients using DPP-4 inhibitors (Table 2). Notably, there were more events of hypoglycemia (BMI <25: 4.7% vs 2.9%; SHR, 1.57; 95% CI, 1.12-2.21; BMI ≥25: 3.4% vs 2.6%; SHR, 1.31; 95% CI, 1.05-1.63) and DKA and HHS (BMI <25: 10.3% vs 7.4%; SHR, 1.39; 95% CI, 1.11-1.74; BMI ≥25: 8.5% vs 6.6%; SHR, 1.30; 95% CI, 1.13-1.50) in the GLP-1 RAs group across all BMI categories.

### Additional and Sensitivity Analysis

**Figure 3.**
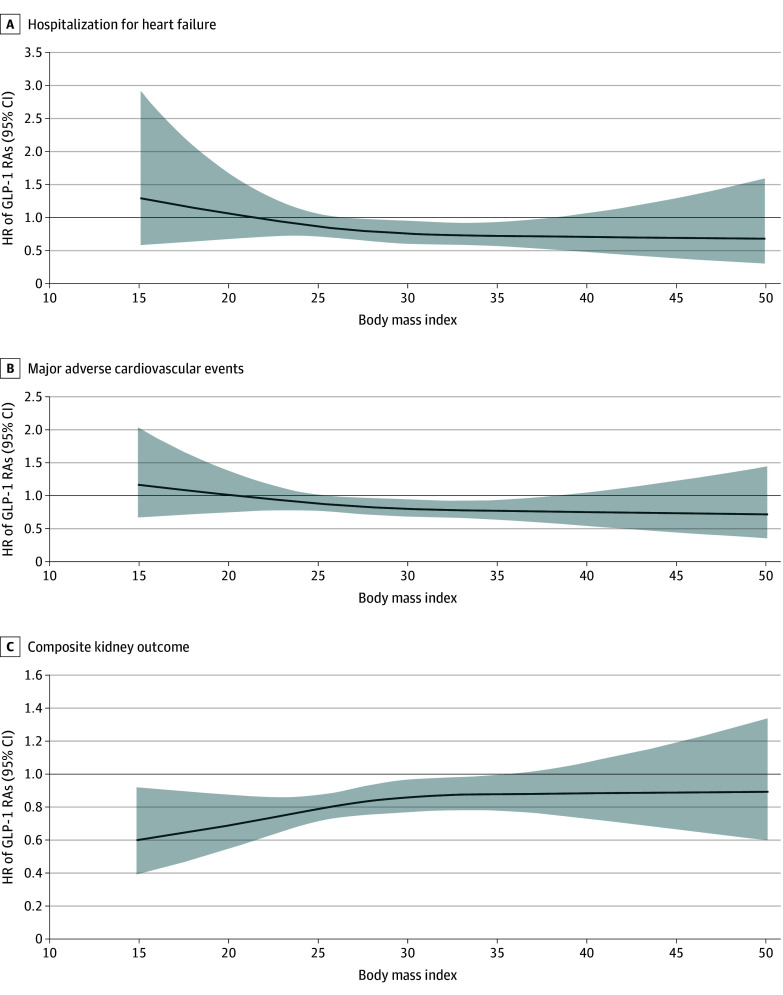
Restricted Cubic Spline Analysis of Body Mass Index Thresholds for Optimal Benefits From Glucagon-Like Peptide-1 Receptor Agonists (GLP-1 RAs) Body mass index is calculated as weight in kilograms divided by height in meters squared. HR indicates hazard ratio.

Several subgroup and sensitivity analyses were conducted to assess the robustness of the main findings. The association of GLP-1 RAs with HHF and MACE was more pronounced among patients with higher BMI, as demonstrated by restricted cubic spline modeling (Figure 3), while kidney benefits were consistent across BMI values. Details on variable distributions, spline modeling, subgroup interactions, propensity score weighting, and complete-case vs imputed analyses are provided in the eResults, eFigures 2 to 5, and eTables 3 to 11 in Supplement 1.

## Discussion

To our knowledge, this cohort study is the first study focusing on the differential outcomes associated with GLP-1 RAs based on BMI. In this large retrospective cohort study, we found that GLP-1 RAs were associated with significantly lower CV risk in patients with type 2 diabetes and BMI 25 or more compared with matched patients using DPP-4 inhibitors. Meanwhile, the protective associations of GLP-1 RAs for kidney outcomes were consistent across all BMI categories. These findings suggest a potential interaction between BMI and CV—but not kidney—benefits of GLP-1 RAs.

### CV Benefits in Overweight

Our observations align with the STEP-HFpEF and SELECT trials, which demonstrated CV benefit of semaglutide in individuals with overweight or obesity.^9,10^ The CV benefits of GLP-1 RAs in patients with overweight may be attributed to their multiple mechanisms of action on insulin sensitivity and inflammation. Obesity is closely associated with heightened insulin resistance and chronic inflammation, both well-known risk factors for CV diseases. GLP-1 RAs enhance insulin sensitivity in skeletal muscle and adipose tissue, independent of weight changes.^21,22^ while also facilitating the cross-talk of adipose tissue and liver during glucose metabolism. This results in reduced hepatic fat content and improved hepatic insulin sensitivity.^23^ Additionally, GLP-1 RAs improve adipocyte function by reducing proinflammatory adipokines and increasing insulin-sensitizing adiponectin secretion.^24^ These metabolic improvements are particularly significant in patients with higher BMI, who typically have more severe adipose dysfunction, suggesting a targeted therapeutic effect in obese patients.

The inflammatory component of obesity contributes significantly to CV complications through endothelial dysfunction, atherosclerotic plaque development, and myocardial remodeling.^23,25,26^ GLP-1 RAs demonstrate substantial anti-inflammatory properties,^27^ including reduction of macrophage infiltration in adipose tissue,^28^ decrease in systemic inflammatory markers (eg, C-reactive protein and interleukin-6), and positively modulate immune cell functions, including those of T cells and macrophages.^29,30^ These anti-inflammatory effects of GLP-1 RAs may be particularly beneficial in patients with higher BMI, who typically exhibit more intense inflammatory states. These agents effectively address obesity-associated CV risks by diminishing both endothelial activation and atherogenic modifications in GLP-1 receptor-expressing cells.^31^

Beyond insulin sensitivity and inflammation, GLP-1 RAs provide comprehensive CV protection through multiple pathways. They improve lipid profiles by reducing low-density lipoprotein cholesterol, total cholesterol, and triglycerides.^32^ Moreover, GLP-1 RAs have been consistently associated with clinically meaningful reductions in systolic blood pressure, which are thought to be mediated through mechanisms involving vasodilation and natriuresis.^33^ The weight reduction effects, particularly significant in patients with higher baseline BMI, contribute to CV benefits.^34^ This multitargeted approach creates more effective CV protection than therapies primarily focusing on glycemic control alone.^35^

In contrast, neutral findings in leaner patients may reflect differences in baseline risk and treatment response. Weight loss induced by GLP-1 RA in patients with low baseline BMI might lead to loss of muscle mass, potentially influencing their overall condition.^36^ Additionally, changes in fluid balance induced by GLP-1 RAs could be less tolerated in patients with preexisting cardiac dysfunction.^37^ Although we did not observe harm in this subgroup, these findings suggest the necessity for cautious use of GLP-1 RAs in populations with low BMI, particularly those with preexisting CV conditions. Further research is needed to elucidate the mechanisms underlying the differential effects of GLP-1 RAs across BMI groups, particularly in patients with low BMI and preexisting HF.

### Kidney Outcomes

Our study corroborates the nephroprotective potential of GLP-1 RAs, demonstrating beneficial associations with kidney outcomes in patients with diabetes irrespective of BMI. The differential associations of GLP-1 RAs with CV vs kidney outcomes across different weight groups may arise from various factors. GLP-1 improved kidney outcomes by inducing natriuresis, mitigating glomerular hyperfiltration, and reducing kidney inflammation and fibrosis.^38^ These mechanisms are independent of body weight, which may explain our result. GLP-1 has been identified in the proximal tubules of rat and pig kidneys and the smooth muscle cells of both monkey and human vasculature, suggesting a systemic role in kidney function regulation.^39,40,41^ The ability of GLP-1 to inhibit sodium-hydrogen exchanger pumps in the proximal tubules significantly contributes to blood pressure control, further benefiting kidney health.^38^ Furthermore, GLP-1 RAs improve kidney hemodynamics by inhibiting pathways of glomerular hyperfiltration in patients with diabetes.^42^ The expression of GLP-1 RAs may also reduce diabetes-induced kidney inflammation and fibrosis.^43^ These mechanisms, largely independent of body weight, support the role of GLP-1 RAs in improving kidney outcomes and suggest that their use could be particularly effective in patients with diabetes regardless of their BMI.

Several pivotal clinical trials supported the kidney protective capabilities of GLP-1 RAs. The LEADER study^4^ found that liraglutide significantly reduced the incidence of new-onset persistent macroalbuminuria. Similarly, the REWIND study^3^ reported improvements in new macroalbuminuria, a sustained decline in eGFR of 30% or more, and reductions in the need for chronic kidney replacement therapy. Moreover, the SUSTAIN-6 study^5^ observed that semaglutide effectively decreased persistent macroalbuminuria and doubled serum creatinine, maintained creatinine clearance above critical levels, and reduced the requirement for continuous kidney replacement therapy. A post hoc analysis of the LEADER and SUSTAIN-6 trials showed consistent kidney benefits of liraglutide and semaglutide across various BMI categories, revealing no heterogeneity in treatment effects.^44^ This finding aligns with our results, confirming the broad nephroprotective potential of GLP-1 RAs, which appears to be effective irrespective of patient BMI. However, more research is needed to explore the relationship between BMI and kidney outcomes with GLP-1 RAs treatment.

### Other Outcomes

Beyond their cardiometabolic outcomes, GLP-1 RAs were associated with reduced all-cause mortality and fewer infection-related hospitalizations across both BMI categories in our cohort. These findings suggest potential extraglycemic benefits of GLP-1 RAs. In patients with BMI 25 or more, a reduction in CV mortality was significantly associated with overall survival benefit. However, among those with BMI less than 25, the reduction in all-cause mortality was observed without significant changes in CV death, suggesting that improvements in non-CV outcomes, such as infection-related events. Recent studies showed that GLP-1 RAs may modulate immune responses, protecting organs in patients with sepsis.^45^ The research by Steven et al^46^ using an endotoxic shock model found that liraglutide could attenuate lipopolysaccharide-induced inflammatory pathways, thereby improving vascular function and reducing oxidative stress.^46^ Another study in lipopolysaccharide-induced septic rats indicated that GLP-1 analogs maintain cardiac function and enhance survival rates.^47^ These pleiotropic effects may contribute to the broader clinical benefits observed in our study.

We also observed a modest increase in hypoglycemia risk among patients using GLP-1 RAs, particularly when combined with other hypoglycemic agents. This risk likely reflects synergistic insulinotropic effects and has been reported in prior clinical trials.^48,49^ Careful patient selection and medication adjustment may be necessary, especially in leaner patients or those receiving combination therapies.

Interestingly, our subgroup analysis indicated that patients receiving concomitant insulin therapy appeared to derive greater CV and kidney benefit from GLP-1 RAs. This may be explained by the fact that insulin-treated patients often have longer disease duration and greater cardiometabolic burden, and may therefore derive more benefit from the complementary mechanisms of GLP-1 RAs, including enhanced insulin sensitivity, reduced inflammation, and nephroprotective effects. The results are consistent with previous research suggesting that GLP-1 RAs remain beneficial even in populations with more advanced diabetes with high disease complexity.^18^

### Limitations

This study has several limitations. First, the nonrandomized design introduces risks of selection bias, residual confounding, and reverse causality. Although propensity score matching improved baseline comparability, unmeasured variables, such as lifestyle, socioeconomic status, and cardiac function, may still influence outcomes. Furthermore, our analysis addressed only baseline covariates and did not account for time-varying confounders that may have arisen during the follow-up period, which could have impacted the observed associations. To mitigate early event bias, we excluded patients with follow-up less than 90 days; however, this approach does not fully address reverse causality.

Additionally, as all data were derived solely from the Chang Gung Research Database, our findings may be more representative of the Taiwanese population and may not be generalizable to other populations or health care settings. We also faced challenges with potential miscoding within the database. Efforts to mitigate this included correlating diagnostic codes with drug registration data, such as using direct eGFR measurements to define kidney function rather than relying on potentially inaccurately coded chronic kidney disease stage diagnoses. Additionally, medication adherence was inferred from prescription data, which may not accurately reflect actual usage, introducing another layer of uncertainty.

## Conclusions

In this cohort study of patients with type 2 diabetes, the CV benefits associated with GLP-1 RAs were BMI-dependent, with significant reductions in CV death and heart failure hospitalization observed only in patients with BMI 25 or greater. In contrast, GLP-1 RAs demonstrated consistent kidney protective associations regardless of BMI, reducing the risk of eGFR decline and progression to dialysis in both BMI groups. Furthermore, GLP-1 RAs were associated with reduced all-cause mortality, infection-related hospitalizations, and all-cause hospitalizations across all BMI categories. However, careful monitoring is needed due to the increased risk of hypoglycemia and DKA or HHS events. Future studies should further investigate the mechanistic pathways of GLP-1 RAs, prescribing patterns in diverse populations, and evaluate their efficacy in patients with HF with preserved ejection fraction and lower BMI through randomized clinical trials.

## Notes

Supplement 1.
eMethods.

eResults.

eReferences.
**eTable 1.** *ICD* codes for diseases used in this study **eTable 2.** Baseline characteristics of type II diabetes mellitus patients treated with GLP-1 RAs versus DPP4i in the matched and imputed cohort stratified by the obesity status **eTable 3.** Major outcomes of type II diabetes mellitus patients treated with dulaglutide versus DPP4i in the propensity matched cohort stratified by the obesity status **eTable 4. **Major outcomes of type II diabetes mellitus patients treated with liraglutide versus DPP4i in the propensity matched cohort stratified by the obesity status **eTable 5.** Subgroup analysis by previous use of SGLT2i in the propensity score matched cohort **eTable 6.** Subgroup analysis by concomitant use of insulin in the propensity score matched cohort **eTable 7.** Major outcomes of type II diabetes mellitus patients treated with GLP-1 RAs versus DPP4i in the propensity matched cohort, excluding patients exposed to thiazolidinediones **eTable 8.** Major outcomes of type II diabetes mellitus patients treated with GLP-1 RAs versus DPP4i in the propensity matched cohort, excluding patients who were diagnosed with pancreatitis **eTable 9.** Major outcomes of type II diabetes mellitus patients treated with GLP-1 RAs versus DPP4i in the IPTW-adjusted cohort **eTable 10.** Baseline characteristics of patients with and without any missing data stratified by the obesity status **eTable 11.** Major outcomes among patients with type 2 diabetes mellitus treated with GLP-1 receptor agonists versus DPP-4 inhibitors, analyzed using multivariable covariate adjustment and multiple imputation, with estimates pooled according to Rubin’s rules **eFigure 1.** Body weight and eGFR changes in the propensity score matched cohort **eFigure 2.** Quantile-quantile plot for the distribution of body mass index **eFigure 3.** Quantile-quantile plot for the distribution of estimated glomerular filtration rate **eFigure 4.** Quantile-quantile plot for the distribution of left ventricular ejection fraction **eFigure 5.** Restricted cubic spline analysis of BMI thresholds for optimal benefits from GLP-1 RAs

Supplement 2.
Data Sharing Statement

## References

[1] Khan MAB, Hashim MJ, King JK, Govender RD, Mustafa H, Al Kaabi J. Epidemiology of type 2 diabetes—global burden of disease and forecasted trends. J Epidemiol Glob Health. 2020;10(1):107-111. doi:10.2991/jegh.k.191028.001 32175717 PMC7310804

[2] Iglay K, Hannachi H, Joseph Howie P, . Prevalence and co-prevalence of comorbidities among patients with type 2 diabetes mellitus. Curr Med Res Opin. 2016;32(7):1243-1252. doi:10.1185/03007995.2016.1168291 26986190

[3] Gerstein HC, Colhoun HM, Dagenais GR, ; REWIND Investigators. Dulaglutide and cardiovascular outcomes in type 2 diabetes (REWIND): a double-blind, randomised placebo-controlled trial. Lancet. 2019;394(10193):121-130. doi:10.1016/S0140-6736(19)31149-3 31189511

[4] Marso SP, Daniels GH, Brown-Frandsen K, ; LEADER Steering Committee; LEADER Trial Investigators. Liraglutide and cardiovascular outcomes in type 2 diabetes. N Engl J Med. 2016;375(4):311-322. doi:10.1056/NEJMoa1603827 27295427 PMC4985288

[5] Marso SP, Bain SC, Consoli A, ; SUSTAIN-6 Investigators. Semaglutide and cardiovascular outcomes in patients with type 2 diabetes. N Engl J Med. 2016;375(19):1834-1844. doi:10.1056/NEJMoa1607141 27633186

[6] Jorsal A, Kistorp C, Holmager P, . Effect of liraglutide, a glucagon-like peptide-1 analogue, on left ventricular function in stable chronic heart failure patients with and without diabetes (LIVE)-a multicentre, double-blind, randomised, placebo-controlled trial. Eur J Heart Fail. 2017;19(1):69-77. doi:10.1002/ejhf.657 27790809

[7] Margulies KB, Hernandez AF, Redfield MM, ; NHLBI Heart Failure Clinical Research Network. Effects of liraglutide on clinical stability among patients with advanced heart failure and reduced ejection fraction: a randomized clinical trial. JAMA. 2016;316(5):500-508. doi:10.1001/jama.2016.10260 27483064 PMC5021525

[8] Neves JS, Packer M, Ferreira JP. Increased risk of heart failure hospitalization with GLP-1 receptor agonists in patients with reduced ejection fraction: a meta-analysis of the EXSCEL and FIGHT trials. J Card Fail. 2023;29(7):1107-1109. doi:10.1016/j.cardfail.2023.03.017 37028749

[9] Kosiborod MN, Abildstrøm SZ, Borlaug BA, ; STEP-HFpEF Trial Committees and Investigators. Semaglutide in patients with heart failure with preserved ejection fraction and obesity. N Engl J Med. 2023;389(12):1069-1084. doi:10.1056/NEJMoa2306963 37622681

[10] Lincoff AM, Brown-Frandsen K, Colhoun HM, ; SELECT Trial Investigators. Semaglutide and cardiovascular outcomes in obesity without diabetes. N Engl J Med. 2023;389(24):2221-2232. doi:10.1056/NEJMoa2307563 37952131

[11] White WB, Cannon CP, Heller SR, ; EXAMINE Investigators. Alogliptin after acute coronary syndrome in patients with type 2 diabetes. N Engl J Med. 2013;369(14):1327-1335. doi:10.1056/NEJMoa1305889 23992602

[12] Green JB, Bethel MA, Armstrong PW, ; TECOS Study Group. Effect of sitagliptin on cardiovascular outcomes in type 2 diabetes. N Engl J Med. 2015;373(3):232-242. doi:10.1056/NEJMoa1501352 26052984

[13] Yoshida K, Solomon DH, Kim SC. Active-comparator design and new-user design in observational studies. Nat Rev Rheumatol. 2015;11(7):437-441. doi:10.1038/nrrheum.2015.30 25800216 PMC4486631

[14] Haam JH, Kim BT, Kim EM, . Diagnosis of obesity: 2022 update of clinical practice guidelines for obesity by the Korean Society for the Study of Obesity. J Obes Metab Syndr. 2023;32(2):121-129. doi:10.7570/jomes23031 37386771 PMC10327686

[15] Ogawa W, Hirota Y, Miyazaki S, ; Creation Committee for Guidelines for the Management of Obesity Disease 2022 by Japan Society for the Study of Obesity (JASSO). Definition, criteria, and core concepts of guidelines for the management of obesity disease in Japan. Endocr J. 2024;71(3):223-231. doi:10.1507/endocrj.EJ23-0593 38123337

[16] Tsai MS, Lin MH, Lee CP, . Chang Gung Research Database: a multi-institutional database consisting of original medical records. Biomed J. 2017;40(5):263-269. doi:10.1016/j.bj.2017.08.002 29179881 PMC6138604

[17] Shao SC, Chan YY, Kao Yang YH, . The Chang Gung Research Database—a multi-institutional electronic medical records database for real-world epidemiological studies in Taiwan. Pharmacoepidemiol Drug Saf. 2019;28(5):593-600. doi:10.1002/pds.4713 30648314

[18] Chen TH, Tseng CJ, Li YR, . Glucagon-like peptide 1 receptor agonists outperform basal insulin in cardiovascular and renal outcomes for type 2 diabetes mellitus: a retrospective cohort study. Acta Diabetol. Published online January 15, 2025. doi:10.1007/s00592-024-02443-6 39812791

[19] Austin PC. Optimal caliper widths for propensity-score matching when estimating differences in means and differences in proportions in observational studies. Pharm Stat. 2011;10(2):150-161. doi:10.1002/pst.433 20925139 PMC3120982

[20] Fine JP, Gray RJ. A proportional hazards model for the subdistribution of a competing risk. J Am Stat Assoc. 1999;94(446):496-509. doi:10.1080/01621459.1999.10474144

[21] Chadt A, Al-Hasani H. Glucose transporters in adipose tissue, liver, and skeletal muscle in metabolic health and disease. Pflugers Arch. 2020;472(9):1273-1298. doi:10.1007/s00424-020-02417-x 32591906 PMC7462924

[22] Mashayekhi M, Nian H, Mayfield D, . Weight loss-independent effect of liraglutide on insulin sensitivity in individuals with obesity and prediabetes. Diabetes. 2024;73(1):38-50. doi:10.2337/db23-0356 37874653 PMC10784656

[23] Cani PD, Knauf C, Iglesias MA, Drucker DJ, Delzenne NM, Burcelin R. Improvement of glucose tolerance and hepatic insulin sensitivity by oligofructose requires a functional glucagon-like peptide 1 receptor. Diabetes. 2006;55(5):1484-1490. doi:10.2337/db05-1360 16644709

[24] Simental-Mendía LE, Sánchez-García A, Linden-Torres E, Simental-Mendía M. Impact of glucagon-like peptide-1 receptor agonists on adiponectin concentrations: a meta-analysis of randomized controlled trials. Br J Clin Pharmacol. 2021;87(11):4140-4149. doi:10.1111/bcp.14855 33835520

[25] Ellulu MS, Patimah I, Khaza’ai H, Rahmat A, Abed Y. Obesity and inflammation: the linking mechanism and the complications. Arch Med Sci. 2017;13(4):851-863. doi:10.5114/aoms.2016.58928 28721154 PMC5507106

[26] Henning RJ. Obesity and obesity-induced inflammatory disease contribute to atherosclerosis: a review of the pathophysiology and treatment of obesity. Am J Cardiovasc Dis. 2021;11(4):504-529. 34548951 PMC8449192

[27] Ma X, Liu Z, Ilyas I, . GLP-1 receptor agonists (GLP-1RAs): cardiovascular actions and therapeutic potential. Int J Biol Sci. 2021;17(8):2050-2068. doi:10.7150/ijbs.59965 34131405 PMC8193264

[28] Lee YS, Park MS, Choung JS, . Glucagon-like peptide-1 inhibits adipose tissue macrophage infiltration and inflammation in an obese mouse model of diabetes. Diabetologia. 2012;55(9):2456-2468. doi:10.1007/s00125-012-2592-3 22722451

[29] Guarnotta V, Bianco MJ, Vigneri E, . Effects of GLP-1 receptor agonists on myokine levels and pro-inflammatory cytokines in patients with type 2 diabetes mellitus. Nutr Metab Cardiovasc Dis. 2021;31(11):3193-3201. doi:10.1016/j.numecd.2021.07.015 34518091

[30] Bendotti G, Montefusco L, Lunati ME, . The anti-inflammatory and immunological properties of GLP-1 receptor agonists. Pharmacol Res. 2022;182:106320. doi:10.1016/j.phrs.2022.106320 35738455

[31] Marx N, Husain M, Lehrke M, Verma S, Sattar N. GLP-1 receptor agonists for the reduction of atherosclerotic cardiovascular risk in patients with type 2 diabetes. Circulation. 2022;146(24):1882-1894. doi:10.1161/CIRCULATIONAHA.122.059595 36508493

[32] Sun F, Wu S, Wang J, . Effect of glucagon-like peptide-1 receptor agonists on lipid profiles among type 2 diabetes: a systematic review and network meta-analysis. Clin Ther. 2015;37(1):225-241.e8. doi:10.1016/j.clinthera.2014.11.008 25554560

[33] Goud A, Zhong J, Peters M, Brook RD, Rajagopalan S. GLP-1 agonists and blood pressure: a review of the evidence. Curr Hypertens Rep. 2016;18(2):16. doi:10.1007/s11906-015-0621-6 26803771

[34] Vilsbøll T, Christensen M, Junker AE, Knop FK, Gluud LL. Effects of glucagon-like peptide-1 receptor agonists on weight loss: systematic review and meta-analyses of randomised controlled trials. BMJ. 2012;344:d7771. doi:10.1136/bmj.d7771 22236411 PMC3256253

[35] Kalofoutis C, Piperi C, Kalofoutis A, Harris F, Phoenix D, Singh J. Type II diabetes mellitus and cardiovascular risk factors: current therapeutic approaches. Exp Clin Cardiol. 2007;12(1):17-28. 18650975 PMC2359621

[36] Carbone S, Billingsley HE, Rodriguez-Miguelez P, . Lean mass abnormalities in heart failure: the role of sarcopenia, sarcopenic obesity, and cachexia. Curr Probl Cardiol. 2020;45(11):100417. doi:10.1016/j.cpcardiol.2019.03.006 31036371 PMC11146283

[37] Gutzwiller JP, Hruz P, Huber AR, . Glucagon-like peptide-1 is involved in sodium and water homeostasis in humans. Digestion. 2006;73(2-3):142-150. doi:10.1159/000094334 16809911

[38] Kim M, Platt MJ, Shibasaki T, . GLP-1 receptor activation and Epac2 link atrial natriuretic peptide secretion to control of blood pressure. Nat Med. 2013;19(5):567-575. doi:10.1038/nm.3128 23542788

[39] Schlatter P, Beglinger C, Drewe J, Gutmann H. Glucagon-like peptide 1 receptor expression in primary porcine proximal tubular cells. Regul Pept. 2007;141(1-3):120-128. doi:10.1016/j.regpep.2006.12.016 17276524

[40] Crajoinas RO, Oricchio FT, Pessoa TD, . Mechanisms mediating the diuretic and natriuretic actions of the incretin hormone glucagon-like peptide-1. Am J Physiol Renal Physiol. 2011;301(2):F355-F363. doi:10.1152/ajprenal.00729.2010 21593184

[41] Pyke C, Heller RS, Kirk RK, . GLP-1 receptor localization in monkey and human tissue: novel distribution revealed with extensively validated monoclonal antibody. Endocrinology. 2014;155(4):1280-1290. doi:10.1210/en.2013-1934 24467746

[42] Skov J. Effects of GLP-1 in the kidney. Rev Endocr Metab Disord. 2014;15(3):197-207. doi:10.1007/s11154-014-9287-7 24791975

[43] Yin W, Xu S, Wang Z, . Recombinant human GLP-1(rhGLP-1) alleviating renal tubulointestitial injury in diabetic STZ-induced rats. Biochem Biophys Res Commun. 2018;495(1):793-800. doi:10.1016/j.bbrc.2017.11.076 29137984

[44] Verma S, McGuire DK, Bain SC, . Effects of glucagon-like peptide-1 receptor agonists liraglutide and semaglutide on cardiovascular and renal outcomes across body mass index categories in type 2 diabetes: results of the LEADER and SUSTAIN 6 trials. Diabetes Obes Metab. 2020;22(12):2487-2492. doi:10.1111/dom.14160 32744418 PMC7754406

[45] Yang F, Zeng F, Luo X, . GLP-1 receptor: a new target for sepsis. Front Pharmacol. 2021;12:706908. doi:10.3389/fphar.2021.706908 34335269 PMC8316682

[46] Steven S, Hausding M, Kröller-Schön S, . Gliptin and GLP-1 analog treatment improves survival and vascular inflammation/dysfunction in animals with lipopolysaccharide-induced endotoxemia. Basic Res Cardiol. 2015;110(2):6. doi:10.1007/s00395-015-0465-x 25600227

[47] Ku HC, Chen WP, Su MJ. GLP-1 signaling preserves cardiac function in endotoxemic Fischer 344 and DPP4-deficient rats. Naunyn Schmiedebergs Arch Pharmacol. 2010;382(5-6):463-474. doi:10.1007/s00210-010-0559-9 20852989

[48] de Heer J, Holst JJ. Sulfonylurea compounds uncouple the glucose dependence of the insulinotropic effect of glucagon-like peptide 1. Diabetes. 2007;56(2):438-443. doi:10.2337/db06-0738 17259389

[49] Filippatos TD, Panagiotopoulou TV, Elisaf MS. Adverse effects of GLP-1 receptor agonists. Rev Diabet Stud. 2014;11(3-4):202-230. doi:10.1900/RDS.2014.11.202 26177483 PMC5397288

