# Supplementary material for: GLP-1 RAs and Cardiovascular and Kidney Outcomes by Body Mass Index in Type 2 Diabetes
Source: JAMA Netw Open. 2025 Sep 8;8(9):e2530952. doi: 10.1001/jamanetworkopen.2025.30952 (PMC12418133; doi:10.1001/jamanetworkopen.2025.30952)
Supplement: Supplement 1. — eMethods. eResults. eReferences. eTable 1. ICD codes for diseases used in this study eTable 2. Baseline characteristics of type II diabetes mellitus patients treated with GLP-1 RAs versus DPP4i in the matched and imputed cohort stratified by the obesity status eTable 3. Major outcomes of type II diabetes mellitus patients treated with dulaglutide versus DPP4i in the propensity matched cohort stratified by the obesity status eTable 4. Major outcomes of type II diabetes mellitus patients treated with liraglutide versus DPP4i in the propensity matched cohort stratified by the obesity status eTable 5. Subgroup analysis by previous use of SGLT2i in the propensity score matched cohort eTable 6. Subgroup analysis by concomitant use of insulin in the propensity score matched cohort eTable 7. Major outcomes of type II diabetes mellitus patients treated with GLP-1 RAs versus DPP4i in the propensity matched cohort, excluding patients exposed to thiazolidinediones eTable 8. Major outcomes of type II diabetes mellitus patients treated with GLP-1 RAs versus DPP4i in the propensity matched cohort, excluding patients who were diagnosed with pancreatitis eTable 9. Major outcomes of type II diabetes mellitus patients treated with GLP-1 RAs versus DPP4i in the IPTW-adjusted cohort eTable 10. Baseline characteristics of patients with and without any missing data stratified by the obesity status eTable 11. Major outcomes among patients with type 2 diabetes mellitus treated with GLP-1 receptor agonists versus DPP-4 inhibitors, analyzed using multivariable covariate adjustment and multiple imputation, with estimates pooled according to Rubin’s rules eFigure 1. Body weight and eGFR changes in the propensity score matched cohort eFigure 2. Quantile-quantile plot for the distribution of body mass index eFigure 3. Quantile-quantile plot for the distribution of estimated glomerular filtration rate eFigure 4. Quantile-quantile plot for the distribution of left ventricular ejection fraction eFig [file jamanetwopen-e2530952-s001.pdf]

Supplemental Online Content

Chen TH, Hu EH, Chen DY, et al. GLP-1 RAs and cardiovascular and kidney outcomes by body mass index in type 2 diabetes. JAMA Netw Open. 2025;8(9):e2530952. doi:10.1001/jamanetworkopen.2025.30952

eMethods.
eResults.
eReferences.
eTable 1. ICD codes for diseases used in this study
eTable 2. Baseline characteristics of type II diabetes mellitus patients treated with GLP-1 RAs versus DPP4i in the matched and imputed cohort stratified by the obesity status
eTable 3. Major outcomes of type II diabetes mellitus patients treated with dulaglutide versus DPP4i in the propensity matched cohort stratified by the obesity status
eTable 4. Major outcomes of type II diabetes mellitus patients treated with liraglutide versus DPP4i in the propensity matched cohort stratified by the obesity status
eTable 5. Subgroup analysis by previous use of SGLT2i in the propensity score matched cohort
eTable 6. Subgroup analysis by concomitant use of insulin in the propensity score matched cohort
eTable 7. Major outcomes of type II diabetes mellitus patients treated with GLP-1 RAs versus DPP4i in the propensity matched cohort, excluding patients exposed to thiazolidinediones
eTable 8. Major outcomes of type II diabetes mellitus patients treated with GLP-1 RAs versus DPP4i in the propensity matched cohort, excluding patients who were diagnosed with pancreatitis
eTable 9. Major outcomes of type II diabetes mellitus patients treated with GLP-1 RAs versus DPP4i in the IPTW-adjusted cohort
eTable 10. Baseline characteristics of patients with and without any missing data stratified by the obesity status
eTable 11. Major outcomes among patients with type 2 diabetes mellitus treated with GLP-1 receptor agonists versus DPP-4 inhibitors, analyzed using

© 2025 Chen TH et al. *JAMA Network Open*.

multivariable covariate adjustment and multiple imputation, with estimates pooled according to Rubin's rules
eFigure 1. Body weight and eGFR changes in the propensity score matched cohort
eFigure 2. Quantile-quantile plot for the distribution of body mass index
eFigure 3. Quantile-quantile plot for the distribution of estimated glomerular filtration rate
eFigure 4. Quantile-quantile plot for the distribution of left ventricular ejection fraction
eFigure 5. Restricted cubic spline analysis of BMI thresholds for optimal benefits from GLP-1 RAs

This supplemental material has been provided by the authors to give readers additional information about their work.

© 2025 Chen TH et al. *JAMA Network Open*.

eMethods

Study Design and Population

The retrospective cohort study using a new-user, active comparator design. The new-user design improves validity by capturing patients at the point of treatment initiation, enabling clearer evaluation of baseline characteristics and time-dependent effects, while also minimizing immortal time bias.[1] The study cohort included patients with T2DM who first received GLP-1 RAs or DPP-4 inhibitors in Chang-Gung Memorial Hospitals from December 15, 2011, to December 31, 2022. Patients were excluded if they were <20 years old, had type 1 diabetes, or had a history of heart failure (HF) with reduced ejection fraction (EF <40%), bariatric surgery, or prior use of sodium-glucose cotransporter 2 (SGLT2) inhibitors. We also excluded patients with missing baseline glycated hemoglobin (HbA1c) or BMI data, follow-up duration <90 days, or drug switching within the first 90 days. According to the recent Asia guidelines for the definition of obesity, we further classified patients based on a BMI threshold of 25 kg/m^2.[2,3] Patients were stratified by BMI (≥25 or <25 kg/m^2) and treatment group (GLP-1 RAs vs DPP-4 inhibitors).

Data source

Databases were searched from the Chang Gung Research Database (CGRD), a de-identified database managed by the Chang Gung Medical Foundation (CGMF)

© 2025 Chen TH et al. *JAMA Network Open*.

healthcare system, the largest healthcare provider in Taiwan. The CGRD, including two medical centers and five general hospitals from northern to southern Taiwan, is Taiwan's largest multi-institutional electronic medical record database. The Institutional Review Board of CGMF approved the study protocol (202302072B0) and waived the requirement for informed consent. Patients' records were anonymized and de-identified before analysis. Details regarding the CGRD have been published elsewhere.[4,5] Diseases were coded using only the International Classification of Diseases, Ninth Revision, Clinical Modification (ICD-9-CM) diagnostic codes for diagnoses made before 2015, and both ICD-9-CM and International Classification of Diseases, Tenth Revision, Clinical Modification (ICD-10-CM) diagnostic codes for diagnoses afterward.

Covariates and Baseline Characteristics

The baseline characteristics included demographics, established atherosclerotic cardiovascular disease (ASCVD), multiple risk factors for ASCVD, severity of diabetes mellitus (DM), left ventricular ejection fraction (LVEF), history of previous HHF, estimated glomerular filtration rate (eGFR) stages, baseline comorbidities, Charlson's Comorbidity Index score, baseline vital signs, biochemistry data, and concomitant medication. Covariates were selected based on clinical relevance and evidence from

© 2025 Chen TH et al. *JAMA Network Open*.

prior literature on established cardiorenal risk factors.[6] Demographic data included age, sex, smoking, alcohol consumption, and body mass index (BMI). The severity of DM was differentiated by the duration of DM, HbA1c level, DM nephropathy, DM retinopathy, DM neuropathy, DM foot, and DM outpatient department follow-up times. We categorized the eGFR stage as ≥60, 30-59, <30, and dialysis. The baseline comorbidities comprised hypertension, dyslipidemia, coronary artery disease, ischemic stroke, myocardial infarction, coronary intervention, peripheral artery disease, critical limb ischemia, atrial fibrillation, gout, pancreatitis, and malignancy. Vital signs consisted of systolic and diastolic blood pressure and heart rate. Biochemistry data included low-density lipoprotein, high-density lipoprotein, total cholesterol, triglyceride, hemoglobin, uric acid, and urine albumin to creatinine ratio (UACR). Concomitant oral agents were composed of anti-glycemic agents (biguanide, sulfonylurea, thiazolidinedione, alpha-glucosidase inhibitors, glinide, and insulin) and cardiovascular agents (lipid-lowering agents, antihypertensive agents, and antiplatelet agents). The ICD diagnostic codes for disease were provided in **Supplement eTable 1.**

Outcomes definition

Clinical outcomes were categorized into CV, renal, and secondary outcomes. **(Table 2)** Our primary outcomes were MACE and renal outcomes. MACE included CV death,

© 2025 Chen TH et al. *JAMA Network Open*.

myocardial infarction, ischemic stroke, and HHF. HHF is defined as any hospital admission with a primary diagnosis of heart failure. The renal outcomes included a decline of eGFR > 50% or progression to dialysis during follow-up. Our secondary outcome included all-cause death, admission due to infection, admission due to any cause, composite MALE (major adverse lower-limb event) outcome, hypoglycemia (fasting glucose <54 mg/dL), DKA/HHS, and a new diagnosis of pancreatitis. Myocardial infarction, ischemic stroke, and HHF were detected in the inpatient setting. Information about the main cause and date of mortality can be linked to the Taiwanese Death Registry database. CV deaths included acute myocardial infarction, sudden cardiac death, heart failure, stroke, cardiovascular procedures, cardiovascular hemorrhage, and related causes. Patients were followed from the date of initial dispensation (the index date) until the day of outcome occurrence, drug switch (e.g., DPP4i to GLP-1 RAs), death, or the end of the database (December 31, 2022), whichever came first.

Statistical analysis

We created a propensity score matching to reduce confounding when comparing the outcomes between treatment groups. All of the variables listed in **Table 1** were included in the propensity score calculation (the follow up duration was replaced with

© 2025 Chen TH et al. *JAMA Network Open*.

the index date), which was derived from a multivariable logistic regression model without interaction terms. We used a greedy nearest neighbor matching algorithm with a caliper of 0.2 times the standard deviation of the logit of the propensity score to ensure close matches while minimizing bias. This caliper width is commonly recommended in the literature as it balances the trade-off between matching quality and sample retention, reducing the risk of poor matches that could distort treatment effect estimates.[7] Matching was conducted in a random order without replacement to ensure unbiased pair selection. Matching was performed separately for the non-obese and obese groups. We examined the balance of covariates by assessing the absolute value of standardized differences in all baseline characteristics between the groups. An absolute value of standardized difference of <0.1 was considered to indicate a negligible between-group difference. The magnitude of the standardized difference is not influenced by sample size, making it a stable measure for assessing baseline covariate balance between groups. Due to the presence of missing values in the covariates, we performed single imputation using the expectation-maximization (EM) algorithm prior to matching. The EM algorithm assumes multivariate normality and missing at random, and iteratively applies an E-step to estimate missing values and an M-step to maximize the likelihood for unbiased parameter estimation.

© 2025 Chen TH et al. *JAMA Network Open*.

All outcome comparisons were made in the propensity score-matched cohort. We applied the Cox proportional hazards model for CV and all-cause deaths. The incidence of nonfatal outcomes (e.g., composite renal outcomes and MALEs) was compared between the groups using the Fine and Gray subdistribution hazards model with considering mortality as a competing risk.[8] We accounted for within-pair clustering of outcomes after matching by using a robust standard error. We further investigated the potential modification effect of BMI on the association between GLP-1 RAs and risks of HHF, MACE, and composite renal outcome. Since the cutoff for defining obesity may be somewhat arbitrary (in our study, obesity was defined as BMI ≥25), this categorization may oversimplify the information contained in BMI as a continuous variable. Therefore, continuous BMI was modeled as a restricted cubic spline (RCS) with knots at the 10th, 50th, and 90th percentiles, allowing the calculation of hazard ratios (HR) for GLP-1 RAs vs. DPP4 inhibitors and their 95% confidence intervals (CI) across the entire BMI range. An alternative set of knots was specified at the 5th, 35th, 65th, and 95th percentiles to assess the robustness of the spline model. The RCS analysis was carried out using R version 4.3.2 with the “rms” and “interactionRCS” packages. The remaining analyses (including matching) were conducted using SAS software (version 9.4; SAS Institute, Cary, NC, USA). Detailed examples of the main analyses can be found in the Supplementary Materials. A 2-sided P <0.05 was

© 2025 Chen TH et al. *JAMA Network Open*.

considered statistically significant.

Additional and sensitivity analyses

To test the robustness of the main results, a series of additional and sensitivity analyses were carried out. First, given that liraglutide and dulaglutide accounted for the majority of GLP-1RA use, we performed propensity score matching and compared outcomes between each individual GLP-1RA (liraglutide and dulaglutide) and DPP-4 inhibitors. Second, patients with previous use of SGLT2 inhibitors were re-included, and this factor was incorporated into the matching process. Outcome comparisons were then conducted stratified by prior SGLT2 inhibitor use. Third, to evaluate the potential modifying effect of insulin, outcome comparisons were stratified according to concomitant insulin use. Fourth, we conducted a sensitivity analysis in which patients with exposure to thiazolidinediones (TZDs) were excluded. Fifth, a sensitivity analysis excluding patients with pancreatitis was conducted, given that pancreatitis is a relative contraindication to GLP-1RA therapy. Sixth, in the primary analysis, each GLP-1RA patient was matched to only one DPP-4i patient, which utilized only a small fraction of the available DPP-4i population. Therefore, we conducted a sensitivity analysis using inverse probability of treatment weighting (IPTW) based on propensity scores, with the estimand set to the average treatment effect on the treated (ATT).

© 2025 Chen TH et al. *JAMA Network Open*.

The IPTW approach made use of data from all patients. Seventh, to account for missing data, we conducted a sensitivity analysis using the multiple imputation method with 10 datasets, pooling the estimates (with adjustment of all the covariates used in calculating propensity score) using Rubin’s rules. The aforementioned analyses focused on three major outcomes of interest: HHF, MACE, and the composite renal outcome.

eResults

Additional and sensitivity analysis

The distributions of key continuous variables were provided for each of the four combinations of non-obese/obese and GLP-1 RAs/DPP4i, including BMI, eGFR, and LVEF (**Supplement eFigure 2 to 4**). The RCS interaction analysis (with 3 knots) demonstrated that the observed benefit of GLP-1 RAs on HHF and MACE was more pronounced in patients with larger BMI values (**Figure 3A to 3B**). In contrast, the benefit of GLP-1 RAs on the composite renal outcome was consistent across all BMI values but appeared more pronounced in patients with lower BMI (**Figure 3C**). The results with 4 knots showed similar findings (**Supplement eFigure 5A to 5C**).

Additional analyses comparing dulaglutide and liraglutide individually to DPP-4

© 2025 Chen TH et al. *JAMA Network Open*.

inhibitors showed associations generally consistent with the primary analysis. However, statistical significance was attenuated in some comparisons due to smaller subgroup sample sizes (**Supplement eTables 3 and 4**). Subgroup analysis by prior SGLT2 inhibitor exposure showed that among patients with obesity, the beneficial effect of GLP-1 RAs on HHF and MACE was less pronounced in those with prior SGLT2i use (*P* for interaction < 0.05) (**Supplement eTable 5**). However, these findings should be interpreted with caution due to the small number of patients with SGLT2i exposure. When stratified by concomitant insulin use, the beneficial effect of GLP-1 RAs appeared more evident among insulin users compared to non-users (all *P* for interaction < 0.1) **(Supplement eTable 6).** Exclusion of patients with prior TZD use yielded consistent effect directions, but statistical significance was attenuated in some outcomes due to reduced sample size, as nearly one-fourth of GLP-1 RA users had concomitant TZD exposure (**Supplement eTable 7**). Excluding patients with pancreatitis also yielded results consistent with the primary analysis (**Supplement eTable 8**). The results based on IPTW were consistent with those based on matching (**Supplement eTable 9**). There were substantial differences in baseline characteristics between patients with complete data and those with missing data, indicating the necessity of conducting a sensitivity analysis to address missingness (**Supplement eTable 10**). Multiple imputation produced hazard ratios similar to those in the primary

© 2025 Chen TH et al. *JAMA Network Open*.

analysis, reinforcing the robustness of the findings and reducing concerns about missing data bias (**Supplement eTable 11**).

© 2025 Chen TH et al. *JAMA Network Open*.

Reference

1. Yoshida K, Solomon DH, Kim SC. Active-comparator design and new-user design in observational studies. *Nat Rev Rheumatol*. Jul 2015;11(7):437-41. doi:10.1038/nrrheum.2015.30
2. Haam JH, Kim BT, Kim EM, et al. Diagnosis of Obesity: 2022 Update of Clinical Practice Guidelines for Obesity by the Korean Society for the Study of Obesity. *J Obes Metab Syndr*. Jun 30 2023;32(2):121-129. doi:10.7570/jomes23031
3. Ogawa W, Hirota Y, Miyazaki S, et al. Definition, criteria, and core concepts of guidelines for the management of obesity disease in Japan. *Endocr J*. Mar 28 2024;71(3):223-231. doi:10.1507/endocrj.EJ23-0593
4. Tsai MS, Lin MH, Lee CP, et al. Chang Gung Research Database: A multi-institutional database consisting of original medical records. *Biomed J*. Oct 2017;40(5):263-269. doi:10.1016/j.bj.2017.08.002
5. Shao SC, Chan YY, Kao Yang YH, et al. The Chang Gung Research Database-A multi-institutional electronic medical records database for real-world epidemiological studies in Taiwan. *Pharmacoepidemiol Drug Saf*. May 2019;28(5):593-600. doi:10.1002/pds.4713
6. Chen TH, Tseng CJ, Li YR, et al. Glucagon-like peptide 1 receptor agonists outperform basal insulin in cardiovascular and renal outcomes for type 2 diabetes mellitus: a retrospective cohort study. *Acta Diabetol*. Jan 15 2025;doi:10.1007/s00592-024-02443-6
7. Austin PC. Optimal caliper widths for propensity-score matching when estimating differences in means and differences in proportions in observational studies. *Pharmaceutical statistics*. 2011;10(2):150-161.
8. Fine JP, Gray RJ. A proportional hazards model for the subdistribution of a competing risk. *Journal of the American statistical association*. 1999;94(446):496-509.

© 2025 Chen TH et al. *JAMA Network Open*.

eTable 1. ICD codes for diseases used in this study

Disease	ICD-9-CM disease code	ICD-10-CM disease code
Diabetes mellitus	250.x	E08–E13
Diabetic nephropathy	250.4x	E112
Diabetic retinopathy	362.0x, 362.1x, 362.2x	E11.31, E11.32, E11.33, E11.34, E11.35
Diabetic neuropathy	250.60, 250.61, 250.62, 250.63, 357.2	E08.42, E09.42, E10.40, E10.42, E10.65, E11.40, E11.42, E11.65, E13.4
Diabetic foot	250.7x	E11.51, E11.52, E11.621, E08.51, E08.52, E10.51, E10.52, E13.51, E13.52
Heart failure	428.x	I50
Hypertension	401.x–405.x	I10–I15, N26.2
Dyslipidemia	272.xx	E77, E780, E781, E782, E783, E784, E785, E786, E881, E753, E755, E882, E756, E789, E7521, E7522, E7524, E7130, E7879, E7881, E7889, E8889, E7870
Coronary artery disease	410.x–414.x	I20–I24
Ischemic stroke	433.x–437.x	I66, I65.1, I65.0, I65.8, I65.9, I63.6, I63.8, I63.9, G45.0, G45.8, G45.1, G45.2, G46.0, G46.1, G46.2, G45.9, G45.4, G46.3, G46.4, G46.5, G46.6, G46.7, G46.8, I67.0, I67.1, I67.2, I67.4, I67.5, I67.6, I67.7, I67.9, I68.0, I68.2, I68.8
Myocardial infarction	410.x, 412.x	I21-I22
Peripheral artery disease	440.x, 441.x, 443.x, 444.0x, 444.8x, 447.8x,	I70, I71, I73, I75, I771, I790, I791, I792,

© 2025 Chen TH et al. *JAMA Network Open*.

Disease	ICD-9-CM disease code	ICD-10-CM disease code
	447.9x, 093.0, 437.3, 444.22, 447.1, 557.1, 557.9, V434	I773, I779, I798, K551, K558, K559, Z958, Z959, I743, I744, I745, I748, I740, I7789
Critical limb ischemia	440.21	I70.211, I70.212, I70.213, I70.218, I70.219
Atrial fibrillation	427.3x	I48
Gout	274.xx	M10, M1A.0, M1A.2, M1A.3, M1A.4, M1A.9, N20.0
Pancreatitis	577.0x, 577.1x	K85.0, K85.1, K85.3, K85.8, K85.9, K86.1
Malignancy	140.xx–208.xx	C00-C96

Abbreviation: ICD-9-CM, International Classification of Diseases, Ninth Revision, Clinical Modification; ICD-10-CM, International Classification of Diseases, Tenth Revision, Clinical Modification.

© 2025 Chen TH et al. *JAMA Network Open*.

eTable 2. Baseline characteristics of type II diabetes mellitus patients treated with GLP-1 RAs versus DPP4i in the matched and imputed cohort stratified by the obesity status

	Non-obese (BMI <25 kg/m^2)			Obese (BMI ≥25 kg/m^2)		
Variable	GLP-1 RAs (*n* = 1,841)	DPP4i (*n* = 1,841)	STD	GLP-1 RAs (*n* = 5,359)	DPP4i (*n* = 5,359)	STD
Demographics						
Age, year	61.2 ± 14.1	61.3 ± 13.5	-0.01	56.1 ± 14.0	56.1 ± 14.3	<0.01
Male	843 (45.8)	840 (45.6)	<0.01	2,630 (49.1)	2,614 (48.8)	0.01
Smoking	287 (15.6)	283 (15.4)	0.01	863 (16.1)	847 (15.8)	0.01
Alcohol consumption	143 (7.8)	148 (8.0)	-0.01	430 (8.0)	407 (7.6)	0.02
Body mass index, kg/m^2	22.7 ± 1.9	22.6 ± 1.9	0.02	30.8 ± 4.7	30.7 ± 5.4	0.01
Established ASCVD†	608 (33.0)	606 (32.9)	<0.01	1,506 (28.1)	1,471 (27.4)	0.01
Multiple risk factors for ASCVD‡	481 (26.1)	504 (27.4)	-0.03	1,133 (21.1)	1,123 (21.0)	<0.01
Severity of diabetes						
Insulin	540 (29.3)	560 (30.4)	-0.02	1,150 (21.5)	1,088 (20.3)	0.03
Diabetes duration, year						
Mean ± standard deviation	8.7 ± 6.7	8.7 ± 6.8	<0.01	7.0 ± 6.0	7.0 ± 6.1	<0.01
Median [25th, 75th percentiles]	8.6 [1.8, 14.9]	8.6 [1.6, 14.9]	NA	6.3 [1.0, 11.6]	6.0 [1.0, 12.1]	NA
Glycated hemoglobin, %	8.7 ± 2.1	8.7 ± 2.2	-0.02	8.6 ± 2.0	8.6 ± 2.0	0.01
Diabetic nephropathy	636 (34.5)	630 (34.2)	0.01	1,603 (29.9)	1,572 (29.3)	0.01
DM retinopathy	390 (21.2)	389 (21.1)	<0.01	793 (14.8)	777 (14.5)	0.01
DM neuropathy	932 (50.6)	930 (50.5)	<0.01	2,253 (42.0)	2,235 (41.7)	0.01
DM foot	92 (5.0)	93 (5.1)	<0.01	163 (3.0)	178 (3.3)	-0.02
No. of outpatient visits for DM						

© 2025 Chen TH et al. *JAMA Network Open*.

Variable	Non-obese (BMI <25 kg/m^2)			Obese (BMI ≥25 kg/m^2)		
	GLP-1 RAs (*n* = 1,841)	DPP4i (*n* = 1,841)	STD	GLP-1 RAs (*n* = 5,359)	DPP4i (*n* = 5,359)	STD
Mean ± standard deviation	5.9 ± 4.9	6.1 ± 5.6	-0.03	5.4 ± 4.4	5.4 ± 4.9	0.01
Median [25th, 75th percentiles]	5.0 [2.0, 9.0]	4.0 [1.0, 9.0]	NA	5.0 [2.0, 8.0]	4.0 [1.0, 7.0]	NA
LVEF, %	68.1 ± 5.5	68.1 ± 5.8	<0.01	68.2 ± 4.9	68.3 ± 4.9	-0.02
<50	33 (6.1)	44 (7.3)	-0.05	54 (3.4)	46 (2.9)	0.03
≥50	511 (93.9)	558 (92.7)	0.05	1,520 (96.6)	1,530 (97.1)	-0.03
HHF in the previous 1 year	50 (2.7)	48 (2.6)	0.01	78 (1.5)	84 (1.6)	-0.01
HHF history	93 (5.1)	88 (4.8)	0.01	214 (4.0)	208 (3.9)	0.01
Serum creatinine, mg/dL	1.2 ± 0.9	1.2 ± 1.0	-0.03	1.1 ± 0.7	1.1 ± 0.7	<0.01
eGFR, ml/min/1.73m^2	83.6 ± 38.8	83.1 ± 40.4	0.01	86.7 ± 36.1	86.8 ± 36.9	<0.01
eGFR stage						
≥60 ml/min/1.73m^2	1,219 (68.4)	1,215 (68.4)	<0.01	3,877 (74.7)	3,874 (74.5)	0.01
30-59 ml/min/1.73m^2	347 (19.5)	331 (18.6)	0.02	902 (17.4)	927 (17.8)	-0.01
<30 ml/min/1.73m^2	154 (8.6)	182 (10.2)	-0.05	320 (6.2)	310 (6.0)	0.01
Dialysis	63 (3.5)	49 (2.8)	0.04	89 (1.7)	91 (1.7)	<0.01
Baseline comorbidity						
Hypertension	1,114 (60.5)	1,130 (61.4)	-0.02	3,790 (70.7)	3,789 (70.7)	<0.01
Dyslipidemia	1,300 (70.6)	1,297 (70.5)	<0.01	3,954 (73.8)	3,950 (73.7)	<0.01
Coronary artery disease	426 (23.1)	410 (22.3)	0.02	1,126 (21.0)	1,099 (20.5)	0.01
Ischemic stroke	156 (8.5)	158 (8.6)	<0.01	291 (5.4)	274 (5.1)	0.01
Myocardial infarction	106 (5.8)	100 (5.4)	0.01	217 (4.0)	196 (3.7)	0.02
Coronary intervention	172 (9.3)	162 (8.8)	0.02	378 (7.1)	356 (6.6)	0.02

© 2025 Chen TH et al. *JAMA Network Open*.

Variable	Non-obese (BMI <25 kg/m^2)			Obese (BMI ≥25 kg/m^2)		
	GLP-1 RAs (*n* = 1,841)	DPP4i (*n* = 1,841)	STD	GLP-1 RAs (*n* = 5,359)	DPP4i (*n* = 5,359)	STD
Peripheral artery disease	142 (7.7)	138 (7.5)	0.01	278 (5.2)	279 (5.2)	<0.01
Critical limb ischemia	49 (2.7)	50 (2.7)	<0.01	71 (1.3)	73 (1.4)	<0.01
Atrial fibrillation	71 (3.9)	76 (4.1)	-0.01	164 (3.1)	154 (2.9)	0.01
Gout	216 (11.7)	214 (11.6)	<0.01	887 (16.6)	928 (17.3)	-0.02
Pancreatitis	53 (2.9)	62 (3.4)	-0.03	132 (2.5)	123 (2.3)	0.01
Malignancy	224 (12.2)	222 (12.1)	<0.01	498 (9.3)	507 (9.5)	-0.01
Charlson's Comorbidity Index score	3.6 ± 2.5	3.6 ± 2.4	-0.02	3.2 ± 2.3	3.2 ± 2.1	<0.01
Baseline vital sign						
Systolic blood pressure, mmHg	135.0 ± 21.1	134.9 ± 21.5	<0.01	141.2 ± 19.8	141.5 ± 20.2	-0.01
Diastolic blood pressure, mmHg	74.5 ± 11.7	74.5 ± 12.1	<0.01	79.4 ± 12.2	79.5 ± 12.7	-0.01
Heart rate, beat/min	85.6 ± 13.6	85.8 ± 14.4	-0.01	86.5 ± 13.5	86.6 ± 14.1	-0.01
Biochemistry data						
Low-density lipoprotein, mg/dL	98.7 ± 43.5	98.9 ± 43.0	<0.01	103.3 ± 49.0	103.0 ± 50.5	0.01
High-density lipoprotein, mg/dL	47.9 ± 13.4	47.9 ± 14.1	-0.01	43.4 ± 10.8	43.5 ± 11.3	-0.01
Total cholesterol, mg/dL	172.4 ± 41.5	173.8 ± 43.0	-0.03	174.7 ± 40.5	174.1 ± 40.3	0.02
Triglyceride, md/dL	157.1 ± 121.1	157.1 ± 118.0	<0.01	194.8 ± 133.5	192.6 ± 135.6	0.02
Hemoglobin, g/dL	12.5 ± 1.7	12.5 ± 2.0	0.03	13.3 ± 1.6	13.3 ± 1.8	<0.01
Uric acid, md/dL	5.5 ± 1.4	5.6 ± 1.4	-0.02	6.1 ± 1.3	6.1 ± 1.4	0.01
UACR, mg/gm						
<30	444 (43.6)	451 (44.1)	-0.01	1,313 (43.8)	1,318 (43.6)	<0.01
30-300	350 (34.3)	333 (32.6)	0.04	1,014 (33.8)	1,062 (35.1)	-0.03

© 2025 Chen TH et al. *JAMA Network Open*.

Variable	Non-obese (BMI <25 kg/m^2) GLP-1 RAs (*n* = 1,841)	DPP4i (*n* = 1,841)	STD	Obese (BMI ≥25 kg/m^2) GLP-1 RAs (*n* = 5,359)	DPP4i (*n* = 5,359)	STD
>300	225 (22.1)	239 (23.4)	-0.03	670 (22.4)	643 (21.3)	0.03
Concomitant oral antiglycemic drugs						
Biguanide (Metformin)	1,135 (61.7)	1,156 (62.8)	-0.02	3,748 (69.9)	3,828 (71.4)	-0.03
Sulfonylurea	1,112 (60.4)	1,171 (63.6)	-0.07	3,222 (60.1)	3,278 (61.2)	-0.02
Thiazolidinedione	388 (21.1)	366 (19.9)	0.03	1,036 (19.3)	1,017 (19.0)	0.01
Alpha glucosidase inhibitors	312 (16.9)	300 (16.3)	0.02	815 (15.2)	796 (14.9)	0.01
Glinide	139 (7.6)	153 (8.3)	-0.03	249 (4.6)	249 (4.6)	<0.01
Insulin	540 (29.3)	560 (30.4)	-0.02	1,150 (21.5)	1,088 (20.3)	0.03
Concomitant cardiovascular agents						
RAASi	808 (43.9)	821 (44.6)	-0.01	3,079 (57.5)	3,085 (57.6)	<0.01
Beta-blocker	387 (21.0)	391 (21.2)	-0.01	1,325 (24.7)	1,322 (24.7)	<0.01
Calcium channel blocker	636 (34.5)	631 (34.3)	0.01	2,345 (43.8)	2,305 (43.0)	0.02
Statin	1,155 (62.7)	1,176 (63.9)	-0.02	3,419 (63.8)	3,423 (63.9)	<0.01
Fibrates	127 (6.9)	107 (5.8)	0.04	641 (12.0)	624 (11.6)	0.01
Aspirin	459 (24.9)	465 (25.3)	-0.01	1,243 (23.2)	1,233 (23.0)	<0.01
P2Y12 receptor blockers	220 (12.0)	215 (11.7)	0.01	369 (6.9)	363 (6.8)	<0.01
Oral anticoagulants	54 (2.9)	55 (3.0)	<0.01	138 (2.6)	131 (2.4)	0.01
Follow-up year						
Mean ± standard deviation	2.6 ± 2.0	2.5 ± 1.9	0.07	3.4 ± 2.5	3.3 ± 2.5	0.03
Median [25th, 75th percentiles]	2.1 [0.9, 3.9]	1.9 [0.9, 3.6]	NA	3.0 [1.1, 4.9]	2.8 [1.2, 4.7]	NA

Abbreviation: BMI, body mass index; GLP-1 RAs, glucagon-like peptide-1 receptor agonists; DPP4i, dipeptidyl peptidase-4 inhibitors; STD, standardized difference; ASCVD, atherosclerotic cardiovascular disease; DM, diabetes mellitus; OPD, outpatient department; LVEF,

© 2025 Chen TH et al. *JAMA Network Open*.

left ventricular ejection fraction; HHF, hospitalization for heart failure; eGFR, estimated glomerular filtration rate; UACR, urine albumin to creatinine ratio; RAASi, renin-angiotensin-aldosterone system inhibitors; P2Y12, purinergic receptor P2Y G-protein coupled 12;

† Any of coronary heart disease, coronary revascularization, ischemic stroke, intracerebral hemorrhage, carotid artery stent, myocardial infarction, peripheral artery disease and lower-limb revascularization;

‡ Male over 55 years old or female over 60 years old with anyone of hyperlipidemia, hypertension and smoke;

Data were presented as frequency (percentage), mean ± standard deviation or median [25th, 75th percentiles].

© 2025 Chen TH et al. *JAMA Network Open*.

eTable 3. Major outcomes of type II diabetes mellitus patients treated with dulaglutide versus DPP4i in the propensity matched cohort stratified by the obesity status

Outcome	Non-obese (BMI <25 kg/m^2)			Obese (BMI ≥25 kg/m^2)		
	Dulaglutide (*n* = 849)	DPP4i (*n* = 849)	HR/SHR (95% CI) of Dulaglutide	Dulaglutide (*n* = 2,324)	DPP4i (*n* = 2,324)	HR/SHR (95% CI) of Dulaglutide
Hospitalization for heart failure	44 (5.2)	37 (4.4)	1.18 (0.76–1.84)	77 (3.3)	103 (4.4)	0.76 (0.56–1.02)
Composite MACE outcome#	87 (10.2)	85 (10.0)	0.999 (0.744–1.341)	169 (7.3)	202 (8.7)	0.84 (0.68–1.02)
Composite renal outcome‡	125 (14.7)	146 (17.2)	0.85 (0.67–1.08)	315 (13.6)	337 (14.5)	0.94 (0.81–1.09)

Abbreviation: DPP4i, dipeptidyl peptidase-4 inhibitors; BMI, body mass index; HR, hazard ratio; SHR, subdistribution hazard ratio; CI, confidence interval; MACE, major adverse cardiovascular events;

# Composite of cardiovascular death, myocardial infarction, ischemic stroke or hospitalization for heart failure;

‡ Any of eGFR decline >50% or progression to dialysis.

eTable 4. Major outcomes of type II diabetes mellitus patients treated with liraglutide versus DPP4i in the propensity matched cohort stratified by the obesity status

Outcome	Non-obese (BMI <25 kg/m^2)			Obese (BMI ≥25 kg/m^2)		
	Liraglutide (*n* = 495)	DPP4i (*n* = 495)	HR/SHR (95% CI) of Liraglutide	Liraglutide (*n* = 2,192)	DPP4i (*n* = 2,192)	HR/SHR (95% CI) of Liraglutide
Hospitalization for heart failure	24 (4.8)	24 (4.8)	0.988 (0.560–1.740)	85 (3.9)	90 (4.1)	0.93 (0.69–1.26)
Composite MACE outcome#	64 (12.9)	50 (10.1)	1.26 (0.87–1.84)	181 (8.3)	187 (8.5)	0.94 (0.76–1.15)
Composite renal outcome‡	85 (17.2)	101 (20.4)	0.83 (0.62–1.11)	393 (17.9)	406 (18.5)	0.93 (0.81–1.07)

Abbreviation: DPP4i, dipeptidyl peptidase-4 inhibitors; BMI, body mass index; HR, hazard ratio; SHR, subdistribution hazard ratio; CI, confidence interval; MACE, major adverse cardiovascular events;

# Composite of cardiovascular death, myocardial infarction, ischemic stroke or hospitalization for heart failure;

‡ Any of eGFR decline >50% or progression to dialysis.

© 2025 Chen TH et al. *JAMA Network Open*.

eTable 5. Subgroup analysis by previous use of SGLT2i (those patients were re-included in the cohort) in the propensity score matched cohort

	Non-obese (BMI <25 kg/m^2)				Obese (BMI ≥25 kg/m^2)			
Outcome / prior use of SGLT2i	GLP-1 RAs (*n* = 2,081)	DPP4i (*n* = 2,081)	HR/SHR (95% CI) of GLP-1 RAs	*P* for interaction	GLP-1 RAs (*n* = 6,086)	DPP4i (*n* = 6,086)	HR/SHR (95% CI) of GLP-1 RAs	*P* for interaction
HHF				0.862				<0.001
No	73 (3.9)	75 (4.0)	0.95 (0.69–1.32)		158 (2.9)	209 (3.9)	0.74 (0.60–0.91)	
Yes	5 (2.4)	5 (2.2)	0.85 (0.24–2.98)		20 (3.1)	3 (0.4)	5.98 (1.78–20.07)	
MACE#				0.220				0.005
No	163 (8.7)	161 (8.7)	0.99 (0.80–1.23)		353 (6.5)	450 (8.3)	0.77 (0.67–0.88)	
Yes	9 (4.3)	13 (5.8)	0.57 (0.24–1.34)		29 (4.4)	13 (1.9)	2.01 (1.05–3.88)	
Renal outcome‡				0.978				0.215
No	234 (12.5)	328 (17.7)	0.68 (0.58–0.81)		736 (13.6)	825 (15.3)	0.86 (0.78–0.95)	
Yes	15 (7.2)	17 (7.5)	0.69 (0.35–1.36)		31 (4.7)	22 (3.2)	1.22 (0.71–2.11)	

Abbreviation: SGLT2i, sodium-glucose cotransporter 2 inhibitors; GLP-1 RAs, glucagon-like peptide-1 receptor agonists; DPP4i, dipeptidyl peptidase-4 inhibitors; BMI, body mass index; HR, hazard ratio; SHR, subdistribution hazard ratio; CI, confidence interval; HHF, hospitalization for heart failure; MACE, major adverse cardiovascular events;

# Composite of cardiovascular death, myocardial infarction, ischemic stroke or hospitalization for heart failure;

‡ Any of eGFR decline >50% or progression to dialysis.

© 2025 Chen TH et al. *JAMA Network Open*.

eTable 6. Subgroup analysis by concomitant use of insulin (those patients with previous use of SGLT2i were re-included in the cohort) in the propensity score matched cohort

	Non-obese (BMI <25 kg/m^2)				Obese (BMI ≥25 kg/m^2)			
Outcome / Use of insulin	GLP-1 RAs (*n* = 2,081)	DPP4i (*n* = 2,081)	HR/SHR (95% CI) of GLP-1 RAs	*P* for interaction	GLP-1 RAs (*n* = 6,086)	DPP4i (*n* = 6,086)	HR/SHR (95% CI) of GLP-1 RAs	*P* for interaction
HHF				0.017				0.090
No	51 (3.5)	38 (2.6)	1.32 (0.86–2.02)		119 (2.5)	127 (2.6)	0.92 (0.72–1.19)	
Yes	27 (4.3)	42 (6.9)	0.60 (0.37–0.98)		59 (4.7)	85 (6.9)	0.64 (0.46–0.90)	
MACE#				0.016				0.100
No	103 (7.1)	84 (5.7)	1.20 (0.90–1.61)		249 (5.2)	282 (5.8)	0.87 (0.73–1.03)	
Yes	69 (11.0)	90 (14.8)	0.71 (0.51–0.97)		133 (10.5)	181 (14.6)	0.68 (0.54–0.86)	
Renal outcome‡				<0.001				<0.001
No	150 (10.3)	159 (10.8)	0.93 (0.74–1.15)		517 (10.7)	518 (10.7)	0.98 (0.86–1.10)	
Yes	99 (15.8)	186 (30.5)	0.45 (0.35–0.58)		250 (19.8)	329 (26.6)	0.65 (0.55–0.77)	

Abbreviation: GLP-1 RAs, glucagon-like peptide-1 receptor agonists; DPP4i, dipeptidyl peptidase-4 inhibitors; BMI, body mass index; HR, hazard ratio; SHR, subdistribution hazard ratio; CI, confidence interval; HHF, hospitalization for heart failure; MACE, major adverse cardiovascular events;

# Composite of cardiovascular death, myocardial infarction, ischemic stroke or hospitalization for heart failure;

‡ Any of eGFR decline >50% or progression to dialysis.

© 2025 Chen TH et al. *JAMA Network Open*.

eTable 7. Major outcomes of type II diabetes mellitus patients treated with GLP-1 RAs versus DPP4i in the propensity matched cohort, excluding patients exposed to thiazolidinediones

	Non-obese (BMI <25 kg/m²)			Obese (BMI ≥25 kg/m²)		
Outcome	GLP-1 RAs (*n* = 1,439)	DPP4i (*n* = 1,439)	HR/SHR (95% CI) of Dulaglutide	GLP-1 RAs (*n* = 4,294)	DPP4i (*n* = 4,294)	HR/SHR (95% CI) of Dulaglutide
Hospitalization for heart failure	56 (3.9)	65 (4.5)	0.85 (0.60–1.22)	139 (3.2)	144 (3.4)	0.97 (0.77–1.23)
Composite MACE outcome#	125 (8.7)	126 (8.8)	0.96 (0.75–1.23)	295 (6.9)	321 (7.5)	0.90 (0.77–1.05)
Composite renal outcome‡	191 (13.3)	238 (16.5)	0.79 (0.65–0.95)*	567 (13.2)	645 (15.0)	0.86 (0.77–0.96)*

Abbreviation: GLP-1 RAs, glucagon-like peptide-1 receptor agonists; DPP4i, dipeptidyl peptidase-4 inhibitors; BMI, body mass index; HR, hazard ratio; SHR, subdistribution hazard ratio; CI, confidence interval; MACE, major adverse cardiovascular events;

# Composite of cardiovascular death, myocardial infarction, ischemic stroke or hospitalization for heart failure;

‡ Any of eGFR decline >50% or progression to dialysis.

eTable 8. Major outcomes of type II diabetes mellitus patients treated with GLP-1 RAs versus DPP4i in the propensity matched cohort, excluding patients who were diagnosed with pancreatitis

	Non-obese (BMI <25 kg/m²)			Obese (BMI ≥25 kg/m²)		
Outcome	GLP-1 RAs (*n* = 1,789)	DPP4i (*n* = 1,789)	HR/SHR (95% CI) of Dulaglutide	GLP-1 RAs (*n* = 5,226)	DPP4i (*n* = 5,226)	HR/SHR (95% CI) of Dulaglutide
Hospitalization for heart failure	71 (4.0)	66 (3.7)	1.05 (0.75–1.48)	154 (2.9)	198 (3.8)	0.78 (0.63–0.96)*
Composite MACE outcome#	156 (8.7)	143 (8.0)	1.03 (0.82–1.30)	335 (6.4)	404 (7.7)	0.82 (0.71–0.94)*
Composite renal outcome‡	218 (12.2)	286 (16.0)	0.72 (0.60–0.86)*	702 (13.4)	743 (14.2)	0.94 (0.85–1.04)

Abbreviation: GLP-1 RAs, glucagon-like peptide-1 receptor agonists; DPP4i, dipeptidyl peptidase-4 inhibitors; BMI, body mass index; HR, hazard ratio; SHR, subdistribution hazard ratio; CI, confidence interval; MACE, major adverse cardiovascular events;

# Composite of cardiovascular death, myocardial infarction, ischemic stroke or hospitalization for heart failure;

‡ Any of eGFR decline >50% or progression to dialysis.

© 2025 Chen TH et al. *JAMA Network Open*.

eTable 9. Major outcomes of type II diabetes mellitus patients treated with GLP-1 RAs versus DPP4i in the IPTW-adjusted cohort

	Non-obese (BMI <25 kg/m^2)			Obese (BMI ≥25 kg/m^2)		
Outcome	GLP-1 RAs (*n* = 1,953.0)	DPP4i (*n* = 1,484.2)	HR/SHR (95% CI) of Dulaglutide	GLP-1 RAs (*n* = 6,332.0)	DPP4i (*n* = 5,508.3)	HR/SHR (95% CI) of Dulaglutide
Hospitalization for heart failure	4.0%	4.1%	1.06 (0.76–1.48)	2.9%	4.4%	0.72 (0.59–0.87)*
Composite MACE outcome#	8.8%	8.8%	1.07 (0.85–1.34)	6.5%	8.9%	0.76 (0.67–0.87)*
Composite renal outcome‡	12.3%	17.2%	0.77 (0.65–0.92)*	13.5%	17.0%	0.84 (0.77–0.92)*

Abbreviation: GLP-1 RAs, glucagon-like peptide-1 receptor agonists; DPP4i, dipeptidyl peptidase-4 inhibitors; IPTW, inverse probability of treatment weighting; BMI, body mass index; HR, hazard ratio; SHR, subdistribution hazard ratio; CI, confidence interval; MACE, major adverse cardiovascular events;

# Composite of cardiovascular death, myocardial infarction, ischemic stroke or hospitalization for heart failure;

‡ Any of eGFR decline >50% or progression to dialysis.

© 2025 Chen TH et al. *JAMA Network Open*.

eTable 10. Baseline characteristics of patients with and without any missing data stratified by the obesity status

Variable	Missing *n* (%)	Non-obese (BMI <25 kg/m^2)			Obese (BMI ≥25 kg/m^2)		
		Complete data (*n* = 2,171)	Missing data (*n* = 37,225)	STD	Complete data (*n* = 3,493)	Missing data (*n* = 54,267)	STD
Demographics							
Age, year	0 (0.0)	65.6 ± 12.5	69.4 ± 11.3	-0.32	61.2 ± 13.2	65.0 ± 12.4	-0.29
Male	0 (0.0)	19,676 (52.9)	1,236 (56.9)	-0.08	29,950 (55.2)	2,055 (58.8)	-0.07
Smoking	0 (0.0)	6,524 (17.5)	471 (21.7)	-0.11	9,274 (17.1)	851 (24.4)	-0.18
Alcohol consumption	0 (0.0)	3,709 (10.0)	272 (12.5)	-0.08	5,419 (10.0)	507 (14.5)	-0.14
Body mass index, kg/m^2	0 (0.0)	22.4 ± 2.0	22.5 ± 1.9	-0.03	29.3 ± 3.9	29.4 ± 4.1	-0.04
Established ASCVD†	0 (0.0)	10,968 (29.5)	1,158 (53.3)	-0.50	16,517 (30.4)	1,922 (55.0)	-0.51
Multiple risk factors for ASCVD‡	0 (0.0)	10,724 (28.8)	719 (33.1)	-0.09	13,733 (25.3)	1,019 (29.2)	-0.09
Severity of diabetes							
Insulin	0 (0.0)	5,812 (15.6)	445 (20.5)	-0.13	6,263 (11.5)	625 (17.9)	-0.18
Diabetes duration, year	0 (0.0)	5.2 ± 5.5	5.5 ± 5.8	-0.06	4.6 ± 5.1	5.0 ± 5.6	-0.07
Glycated hemoglobin, %	0 (0.0)	8.4 ± 2.1	8.0 ± 1.9	0.19	8.4 ± 1.9	8.1 ± 1.8	0.14
Diabetic nephropathy	0 (0.0)	6,584 (17.7)	609 (28.1)	-0.25	9,504 (17.5)	900 (25.8)	-0.20
DM retinopathy	0 (0.0)	3,913 (10.5)	258 (11.9)	-0.04	4,478 (8.3)	392 (11.2)	-0.10
DM neuropathy	0 (0.0)	9,802 (26.3)	680 (31.3)	-0.11	13,052 (24.1)	1,000 (28.6)	-0.10
DM foot	0 (0.0)	1,040 (2.8)	99 (4.6)	-0.09	1,021 (1.9)	103 (2.9)	-0.07
No. of outpatient visits for DM	0 (0.0)	3.8 ± 4.2	4.6 ± 4.9	-0.18	3.8 ± 3.9	4.6 ± 4.9	-0.19
LVEF, %	65,310 (67.2)	67.9 ± 9.8	67.3 ± 9.7	0.06	68.0 ± 9.2	68.0 ± 9.1	<0.01
<50		622 (5.9)	137 (6.3)	-0.02	643 (4.1)	142 (4.1)	<0.01
≥50		9,911 (94.1)	2,034 (93.7)	0.02	15,006 (95.9)	3,351 (95.9)	<0.01

© 2025 Chen TH et al. *JAMA Network Open*.

Variable	Missing n (%)	Non-obese (BMI <25 kg/m^2)			Obese (BMI ≥25 kg/m^2)		
		Complete data (n = 2,171)	Missing data (n = 37,225)	STD	Complete data (n = 3,493)	Missing data (n = 54,267)	STD
HHF in the previous 1 year	0 (0.0)	832 (2.2)	190 (8.8)	-0.29	822 (1.5)	205 (5.9)	-0.23
HHF history	0 (0.0)	1,390 (3.7)	270 (12.4)	-0.32	1,801 (3.3)	368 (10.5)	-0.29
Serum creatinine, mg/dL	4,149 (4.3)	1.1 ± 0.9	1.3 ± 1.0	-0.24	1.1 ± 0.8	1.3 ± 1.0	-0.29
eGFR, ml/min/1.73m^2	4,149 (4.3)	86.5 ± 38.8	72.2 ± 37.9	0.37	85.5 ± 35.4	71.8 ± 35.9	0.38
eGFR stage	2,720 (2.8)						
≥60 ml/min/1.73m^2		26,689 (73.7)	1,275 (58.7)	0.32	39,852 (75.8)	2,111 (60.4)	0.34
30-59 ml/min/1.73m^2		6,275 (17.3)	620 (28.6)	-0.27	9,078 (17.3)	955 (27.3)	-0.24
<30 ml/min/1.73m^2		2,488 (6.9)	276 (12.7)	-0.20	2,961 (5.6)	427 (12.2)	-0.23
Dialysis		775 (2.1)	0 (0.0)	0.21	654 (1.2)	0 (0.0)	0.16
Baseline comorbidity							
Hypertension	0 (0.0)	22,467 (60.4)	1,680 (77.4)	-0.37	38,781 (71.5)	2,958 (84.7)	-0.32
Dyslipidemia	0 (0.0)	19,919 (53.5)	1,234 (56.8)	-0.07	33,821 (62.3)	2,291 (65.6)	-0.07
Coronary artery disease	0 (0.0)	6,553 (17.6)	844 (38.9)	-0.49	11,426 (21.1)	1,497 (42.9)	-0.48
Ischemic stroke	0 (0.0)	3,767 (10.1)	322 (14.8)	-0.14	4,411 (8.1)	459 (13.1)	-0.16
Myocardial infarction	0 (0.0)	1,405 (3.8)	226 (10.4)	-0.26	2,018 (3.7)	332 (9.5)	-0.23
Coronary intervention	0 (0.0)	2,028 (5.4)	363 (16.7)	-0.36	3,251 (6.0)	586 (16.8)	-0.34
Peripheral artery disease	0 (0.0)	1,883 (5.1)	201 (9.3)	-0.16	2,219 (4.1)	245 (7.0)	-0.13
Critical limb ischemia	0 (0.0)	505 (1.4)	64 (2.9)	-0.11	450 (0.8)	54 (1.5)	-0.07
Atrial fibrillation	0 (0.0)	1,542 (4.1)	286 (13.2)	-0.33	2,066 (3.8)	390 (11.2)	-0.28
Gout	0 (0.0)	3,654 (9.8)	346 (15.9)	-0.18	7,888 (14.5)	777 (22.2)	-0.20
Pancreatitis	0 (0.0)	960 (2.6)	66 (3.0)	-0.03	1,062 (2.0)	86 (2.5)	-0.03

© 2025 Chen TH et al. *JAMA Network Open*.

Variable	Missing *n* (%)	Non-obese (BMI <25 kg/m^2)			Obese (BMI ≥25 kg/m^2)		
		Complete data (*n* = 2,171)	Missing data (*n* = 37,225)	STD	Complete data (*n* = 3,493)	Missing data (*n* = 54,267)	STD
Malignancy	0 (0.0)	5,516 (14.8)	457 (21.1)	-0.16	6,120 (11.3)	565 (16.2)	-0.14
Charlson's Comorbidity Index score	0 (0.0)	3.2 ± 2.3	4.3 ± 2.6	-0.45	3.0 ± 2.1	4.0 ± 2.4	-0.45
Baseline vital sign							
Systolic blood pressure, mmHg	1,260 (1.3)	135.8 ± 21.4	134.9 ± 22.6	0.04	141.5 ± 20.1	140.9 ± 21.7	0.03
Diastolic blood pressure, mmHg	1,269 (1.3)	74.8 ± 11.9	73.4 ± 12.4	0.12	79.1 ± 12.2	77.4 ± 12.7	0.13
Heart rate, beat/min	1,398 (1.4)	84.6 ± 14.3	83.7 ± 15.0	0.06	84.3 ± 13.9	83.0 ± 14.3	0.09
Biochemistry data							
Low-density lipoprotein, mg/dL	8,502 (8.8)	102.6 ± 45.1	97.7 ± 45.0	0.11	106.3 ± 51.2	101.0 ± 48.5	0.11
High-density lipoprotein, mg/dL	13,325 (13.7)	46.3 ± 13.7	44.7 ± 13.3	0.11	43.6 ± 11.5	42.7 ± 11.6	0.08
Total cholesterol, mg/dL	9,769 (10.1)	174.5 ± 42.5	168.4 ± 45.4	0.14	177.5 ± 41.2	172.6 ± 43.4	0.11
Triglyceride, md/dL	9,894 (10.2)	148.8 ± 107.1	147.4 ± 105.2	0.01	182.6 ± 127.1	175.5 ± 114.3	0.06
Hemoglobin, g/dL	41,500 (42.7)	12.2 ± 2.2	12.1 ± 2.2	0.05	13.0 ± 2.2	12.9 ± 2.2	0.04
Uric acid, md/dL	46,405 (47.8)	5.8 ± 1.9	6.1 ± 2.0	-0.12	6.2 ± 1.8	6.4 ± 1.9	-0.09
UACR, mg/gm	53,596 (55.2)						
<30		8,665 (58.0)	930 (42.8)	0.31	12,417 (54.1)	1,527 (43.7)	0.21
30-300		4,212 (28.2)	697 (32.1)	-0.09	7,052 (30.7)	1,094 (31.3)	-0.01
>300		2,061 (13.8)	544 (25.1)	-0.29	3,489 (15.2)	872 (25.0)	-0.25
Concomitant oral antiglycemic drugs							
Biguanide (Metformin)	0 (0.0)	29,049 (78.0)	1,571 (72.4)	0.13	44,145 (81.3)	2,546 (72.9)	0.20
Sulfonylurea	0 (0.0)	20,589 (55.3)	1,149 (52.9)	0.05	28,638 (52.8)	1,736 (49.7)	0.06
Thiazolidinedione	0 (0.0)	2,533 (6.8)	155 (7.1)	-0.01	4,749 (8.8)	337 (9.6)	-0.03

© 2025 Chen TH et al. *JAMA Network Open*.

Variable	Missing *n* (%)	Non-obese (BMI <25 kg/m^2) Complete data (*n* = 2,171)	Missing data (*n* = 37,225)	STD	Obese (BMI ≥25 kg/m^2) Complete data (*n* = 3,493)	Missing data (*n* = 54,267)	STD
Alpha glucosidase inhibitors	0 (0.0)	4,308 (11.6)	299 (13.8)	-0.07	5,707 (10.5)	430 (12.3)	-0.06
Glinide	0 (0.0)	2,649 (7.1)	199 (9.2)	-0.08	2,634 (4.9)	222 (6.4)	-0.07
Concomitant cardiovascular agents							
RAASi	0 (0.0)	16,039 (43.1)	1,320 (60.8)	-0.36	30,832 (56.8)	2,524 (72.3)	-0.33
Beta-blocker	0 (0.0)	7,198 (19.3)	846 (39.0)	-0.44	13,375 (24.6)	1,434 (41.1)	-0.35
Calcium channel blocker	0 (0.0)	14,024 (37.7)	1,125 (51.8)	-0.29	25,429 (46.9)	2,151 (61.6)	-0.30
Statin	0 (0.0)	18,330 (49.2)	1,279 (58.9)	-0.20	30,742 (56.6)	2,329 (66.7)	-0.21
Fibrates	0 (0.0)	2,126 (5.7)	138 (6.4)	-0.03	5,266 (9.7)	282 (8.1)	0.06
Aspirin	0 (0.0)	8,937 (24.0)	904 (41.6)	-0.38	14,115 (26.0)	1,535 (43.9)	-0.38
P2Y12 receptor blockers	0 (0.0)	3,191 (8.6)	525 (24.2)	-0.43	3,993 (7.4)	698 (20.0)	-0.37
Oral anticoagulants	0 (0.0)	1,220 (3.3)	239 (11.0)	-0.30	1,683 (3.1)	355 (10.2)	-0.29
Follow-up year	0 (0.0)	4.4 ± 3.0	4.0 ± 2.8	0.15	4.8 ± 3.0	4.2 ± 2.8	0.21

Abbreviation: BMI, body mass index; GLP-1 RAs, glucagon-like peptide-1 receptor agonists; DPP4i, dipeptidyl peptidase-4 inhibitors; STD, standardized difference; ASCVD, atherosclerotic cardiovascular disease; DM, diabetes mellitus; OPD, outpatient department; LVEF, left ventricular ejection fraction; HHF, hospitalization for heart failure; eGFR, estimated glomerular filtration rate; UACR, urine albumin to creatinine ratio; RAASi, renin-angiotensin-aldosterone system inhibitors; P2Y12, purinergic receptor P2Y G-protein coupled 12;

† Any of coronary heart disease, coronary revascularization, ischemic stroke, intracerebral hemorrhage, carotid artery stent, myocardial infarction, peripheral artery disease and lower-limb revascularization;

‡ Male over 55 years old or female over 60 years old with anyone of hyperlipidemia, hypertension and smoke;

Data were presented as frequency (percentage), mean ± standard deviation or median [25th, 75th percentiles].

© 2025 Chen TH et al. *JAMA Network Open*.

eTable 11. Major outcomes among patients with type 2 diabetes mellitus treated with GLP-1 receptor agonists versus DPP-4 inhibitors, analyzed using multivariable covariate adjustment and multiple imputation (10 datasets), with estimates pooled according to Rubin's rules

	Non-obese (BMI <25 kg/m^2)			Obese (BMI ≥25 kg/m^2)		
Outcome	GLP-1 RAs (*n* = 1,953)	DPP4i (*n* = 37,443)	HR/SHR (95% CI) of Dulaglutide	GLP-1 RAs (*n* = 6,332)	DPP4i (*n* = 51,428)	HR/SHR (95% CI) of Dulaglutide
Hospitalization for heart failure	79 (4.0)	1,980 (5.3)	0.89 (0.69–1.14)	185 (2.9)	2,476 (4.8)	0.81 (0.68–0.97)*
Composite MACE outcome#	172 (8.8)	5,223 (13.9)	0.91 (0.78–1.07)	412 (6.5)	6,080 (11.8)	0.86 (0.77–0.96)*
Composite renal outcome‡	241 (12.3)	9,100 (24.3)	0.69 (0.60–0.79)*	857 (13.5)	10,317 (20.1)	0.90 (0.83–0.98)*

Abbreviation: GLP-1 RAs, glucagon-like peptide-1 receptor agonists; DPP4i, dipeptidyl peptidase-4 inhibitors; IPTW, inverse probability of treatment weighting; BMI, body mass index; HR, hazard ratio; SHR, subdistribution hazard ratio; CI, confidence interval; MACE, major adverse cardiovascular events;

# Composite of cardiovascular death, myocardial infarction, ischemic stroke or hospitalization for heart failure;

‡ Any of eGFR decline >50% or progression to dialysis.

© 2025 Chen TH et al. *JAMA Network Open*.

eFigure 1. Body Weight and eGFR Changes in the Propensity Score Matched Cohort

(A) Body weight changes in BMI <25 kg/m^2, (B) Body weight changes in BMI ≥25 kg/m^2, (C) eGFR changes in BMI <25 kg/m^2, (D) eGFR changes in BMI ≥25 kg/m^2. BMI, body mass index; DPP4i, DPP-4 inhibitors; GLP-1 RAs, glucagon-like peptide-1 receptor agonists; eGFR, estimated glomerular filtration rate

A

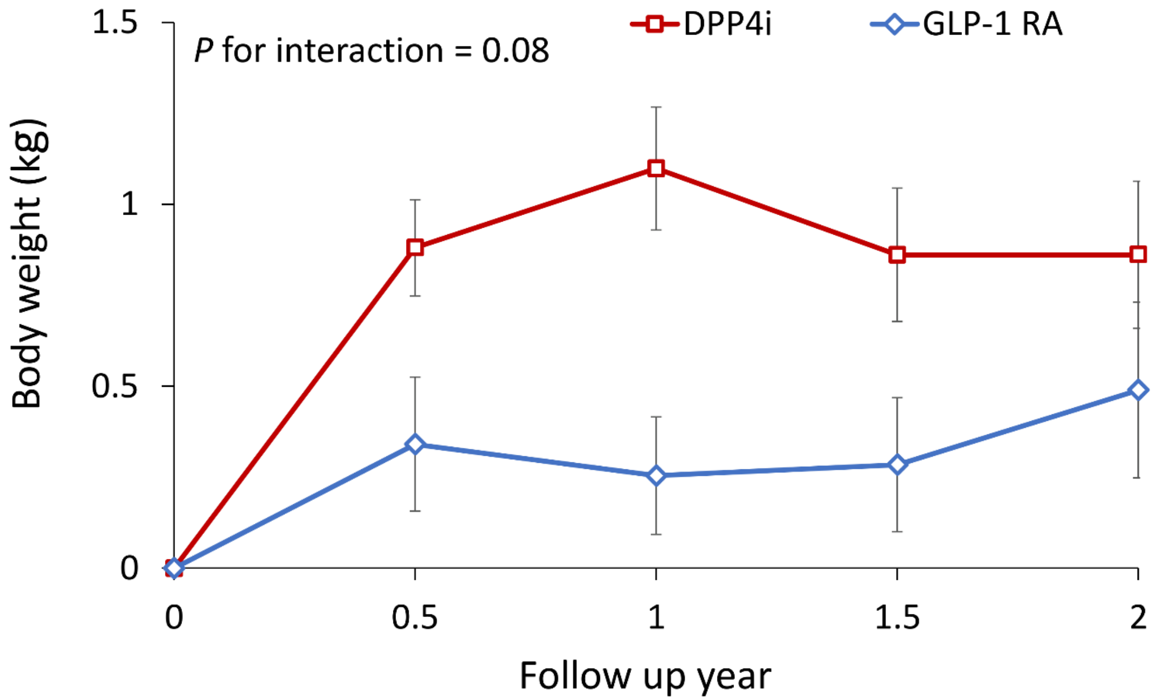

Available number

DPP4i	1775	1141	908	699	586
GLP-1 RA	1815	1209	1013	776	674

© 2025 Chen TH et al. *JAMA Network Open*.

B

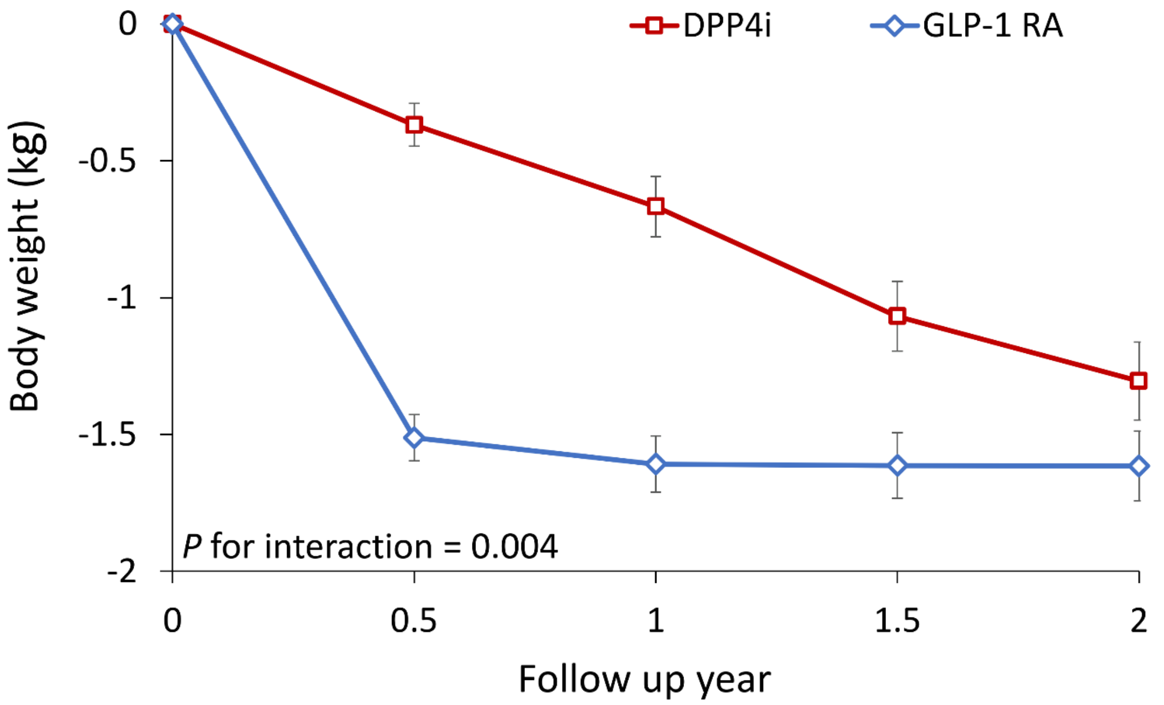

Available number

DPP4i	5222	3447	2864	2357	2104
GLP-1 RA	5318	4056	3419	2811	2602

© 2025 Chen TH et al. *JAMA Network Open*.

C

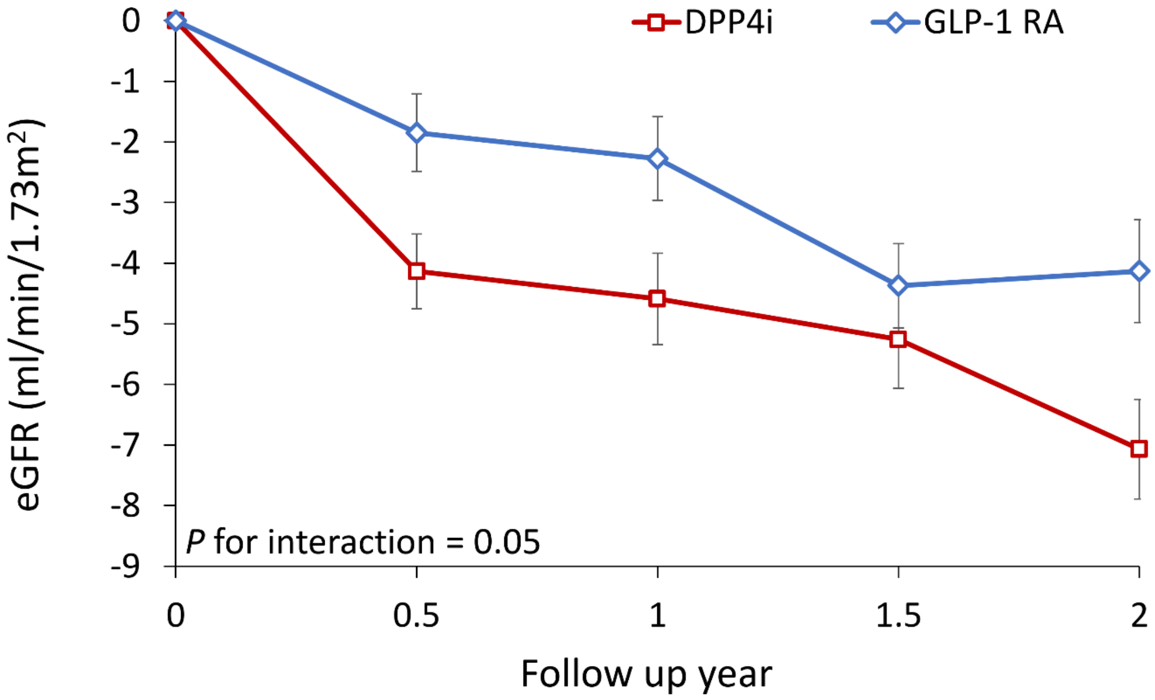

Available number

DPP4i	1666	1394	1120	872	705
GLP-1 RA	1679	1372	1154	913	796

© 2025 Chen TH et al. *JAMA Network Open*.

D

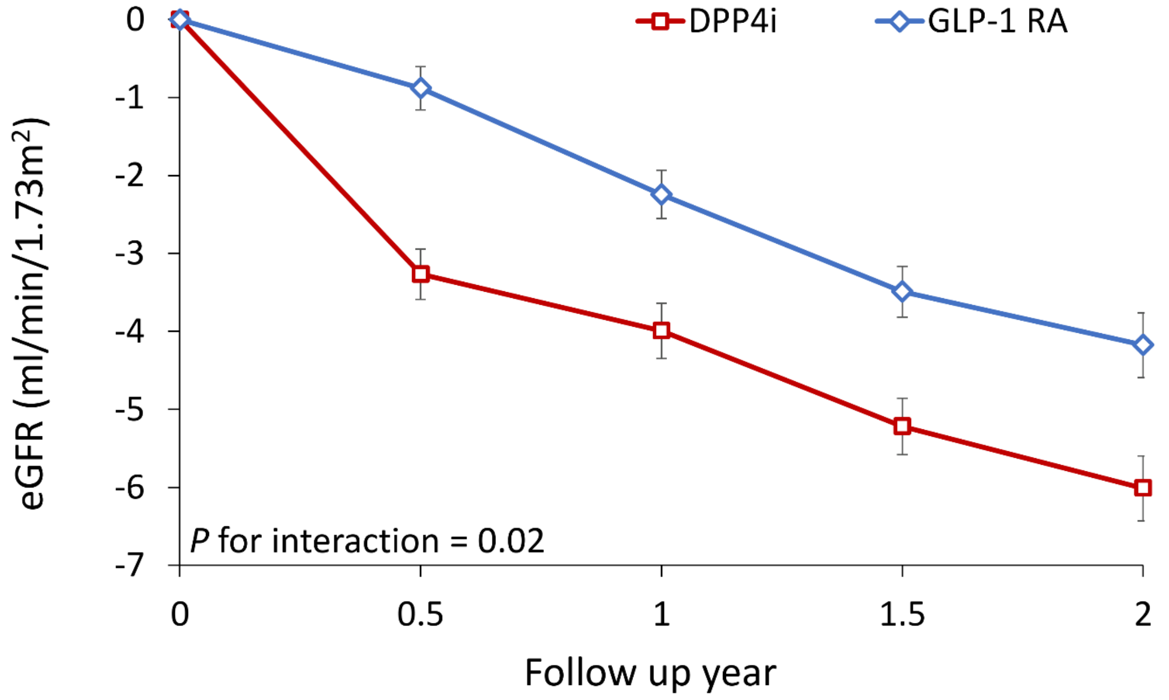

Available number

DPP4i	5029	4185	3585	3028	2652
GLP-1 RA	5020	4194	3635	3073	2840

© 2025 Chen TH et al. *JAMA Network Open*.

eFigure 2. Quantile-Quantile Plot for the Distribution of Body Mass Index

(A) Body weight changes in BMI <25 kg/m^2, (B) Body weight changes in BMI ≥25 kg/m^2, (C) eGFR changes in BMI <25 kg/m^2, (D) eGFR changes in BMI ≥25 kg/m^2. BMI, body mass index; DPP4i, DPP-4 inhibitors; GLP-1 RAs, glucagon-like peptide-1 receptor agonists

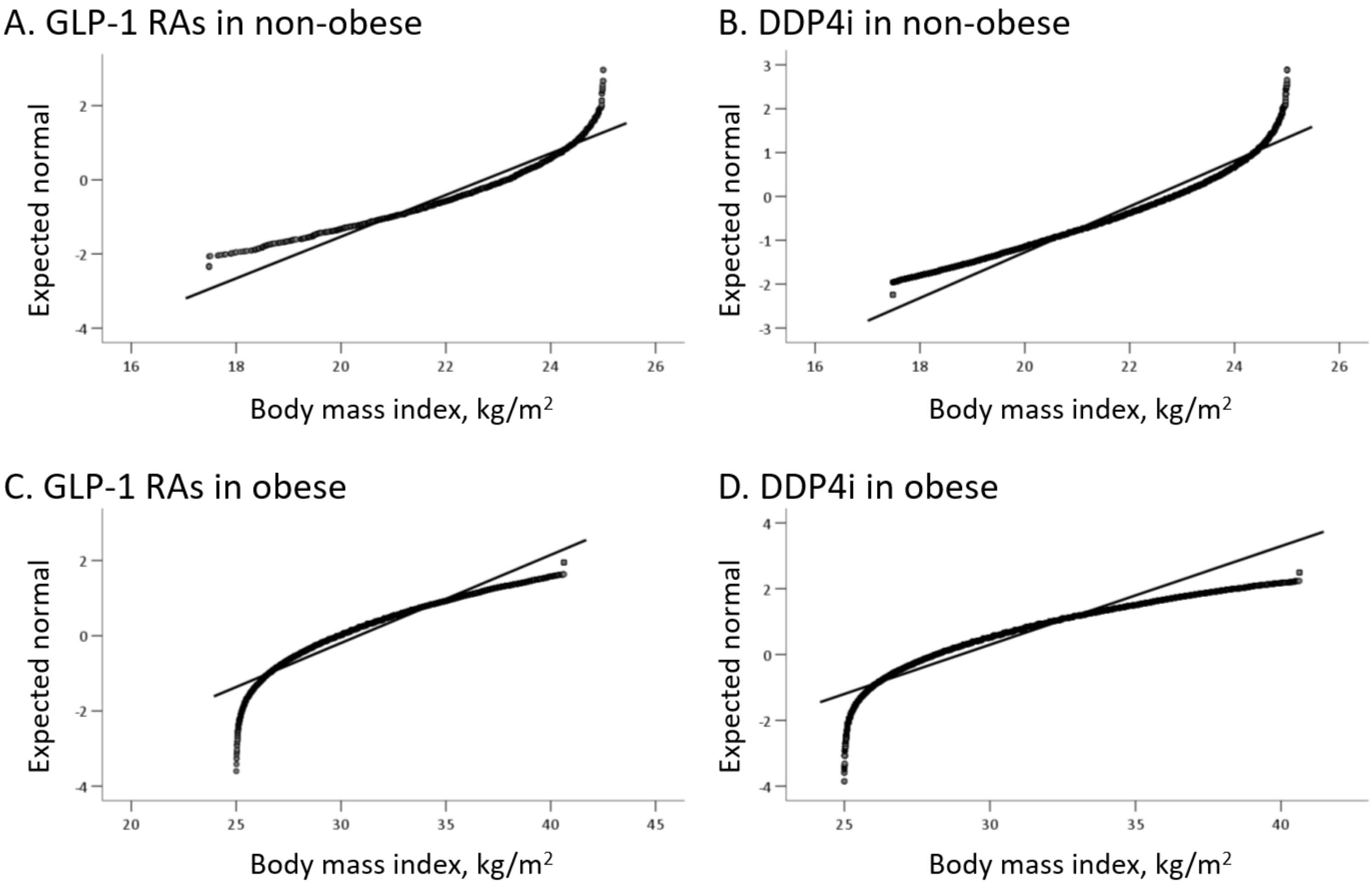

© 2025 Chen TH et al. *JAMA Network Open*.

eFigure 3. Quantile-Quantile Plot for the Distribution of Estimated Glomerular Filtration Rate

(A) Body weight changes in BMI <25 kg/m^2, (B) Body weight changes in BMI ≥25 kg/m^2, (C) eGFR changes in BMI <25 kg/m^2, (D) eGFR changes in BMI ≥25 kg/m^2. BMI, body mass index; DPP4i, DPP-4 inhibitors; GLP-1 RAs, glucagon-like peptide-1 receptor agonists; eGFR, estimated glomerular filtration rate

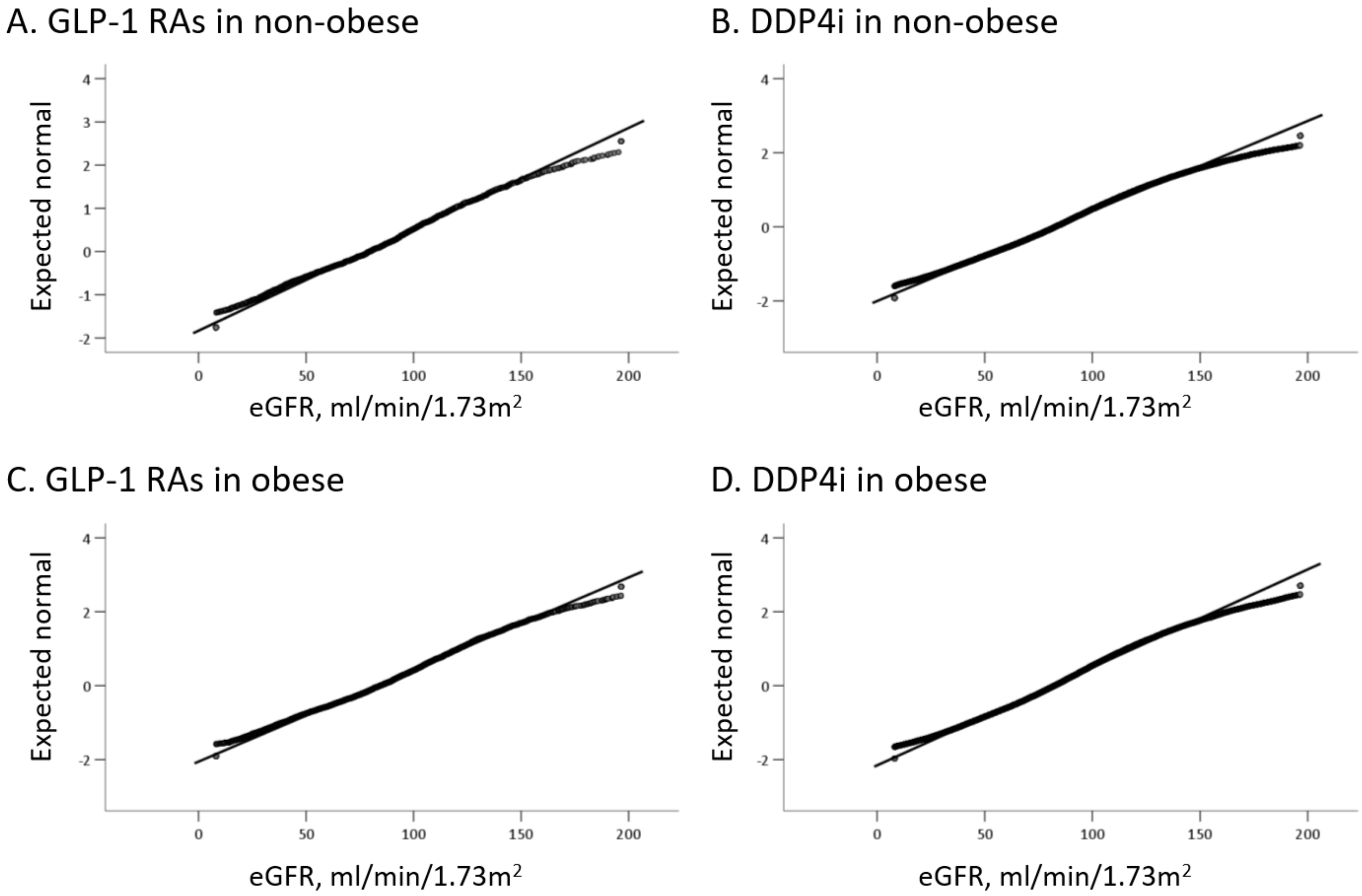

© 2025 Chen TH et al. *JAMA Network Open*.

eFigure 4. Quantile-Quantile Plot for the Distribution of Left Ventricular Ejection Fraction

(A) Body weight changes in BMI <25 kg/m^2, (B) Body weight changes in BMI ≥25 kg/m^2, (C) eGFR changes in BMI <25 kg/m^2, (D) eGFR changes in BMI ≥25 kg/m^2. BMI, body mass index; DPP4i, DPP-4 inhibitors; GLP-1 RAs, glucagon-like peptide-1 receptor agonists; LVEF, left ventricular ejection fraction

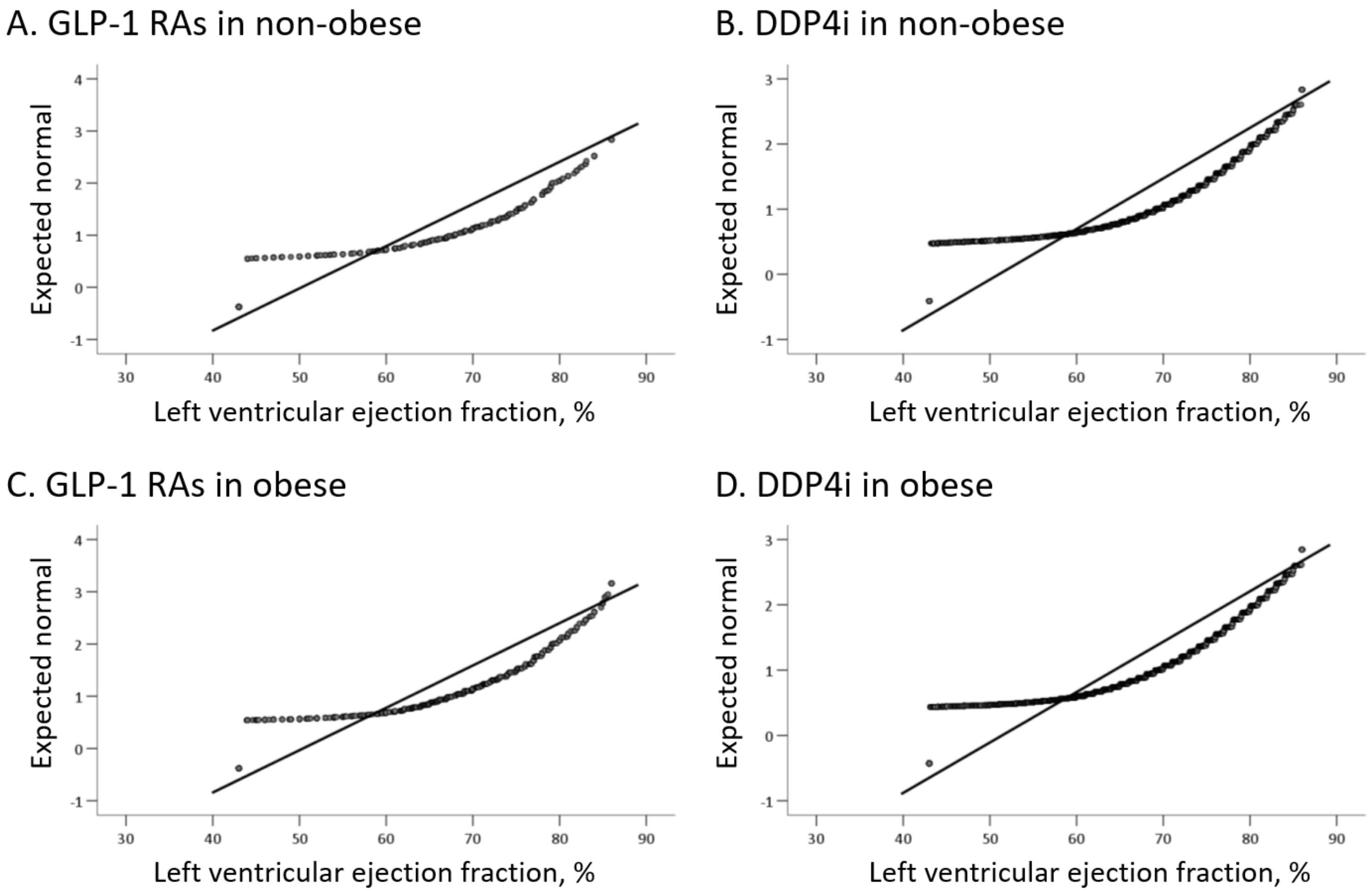

© 2025 Chen TH et al. *JAMA Network Open*.

eFigure 5. Restricted Cubic Spline Analysis of BMI Thresholds for Optimal Benefits from GLP-1 RAs (4 knots)

(A) Hospitalization for heart failure (B) Major adverse cardiovascular events (C) Composite renal outcomes. The shaded area represents the 95% confidence interval of the estimate. BMI, body mass index; DPP4i, DPP-4 inhibitors; GLP-1 RAs, glucagon-like peptide-1 receptor agonists

A. Hospitalization for heart failure

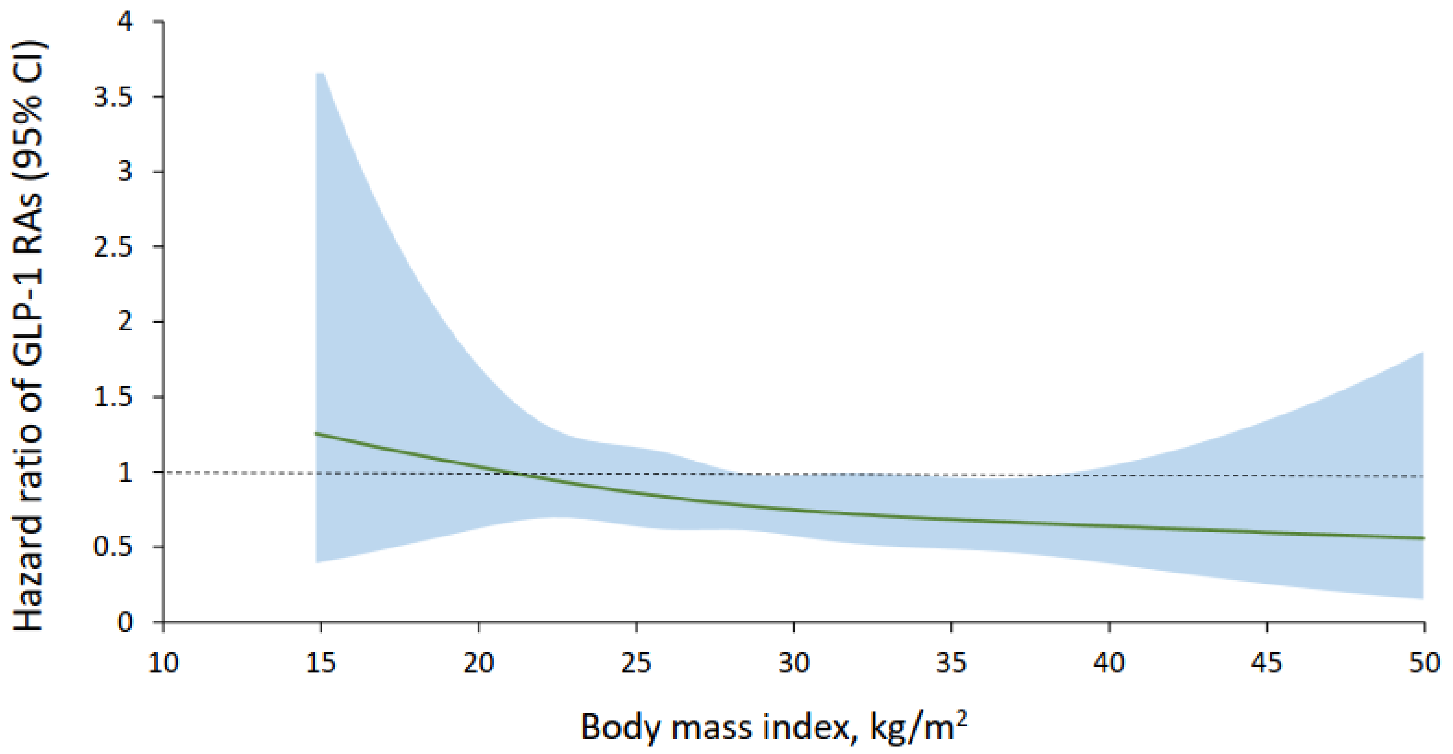

© 2025 Chen TH et al. *JAMA Network Open*.

B. Major adverse cardiovascular events

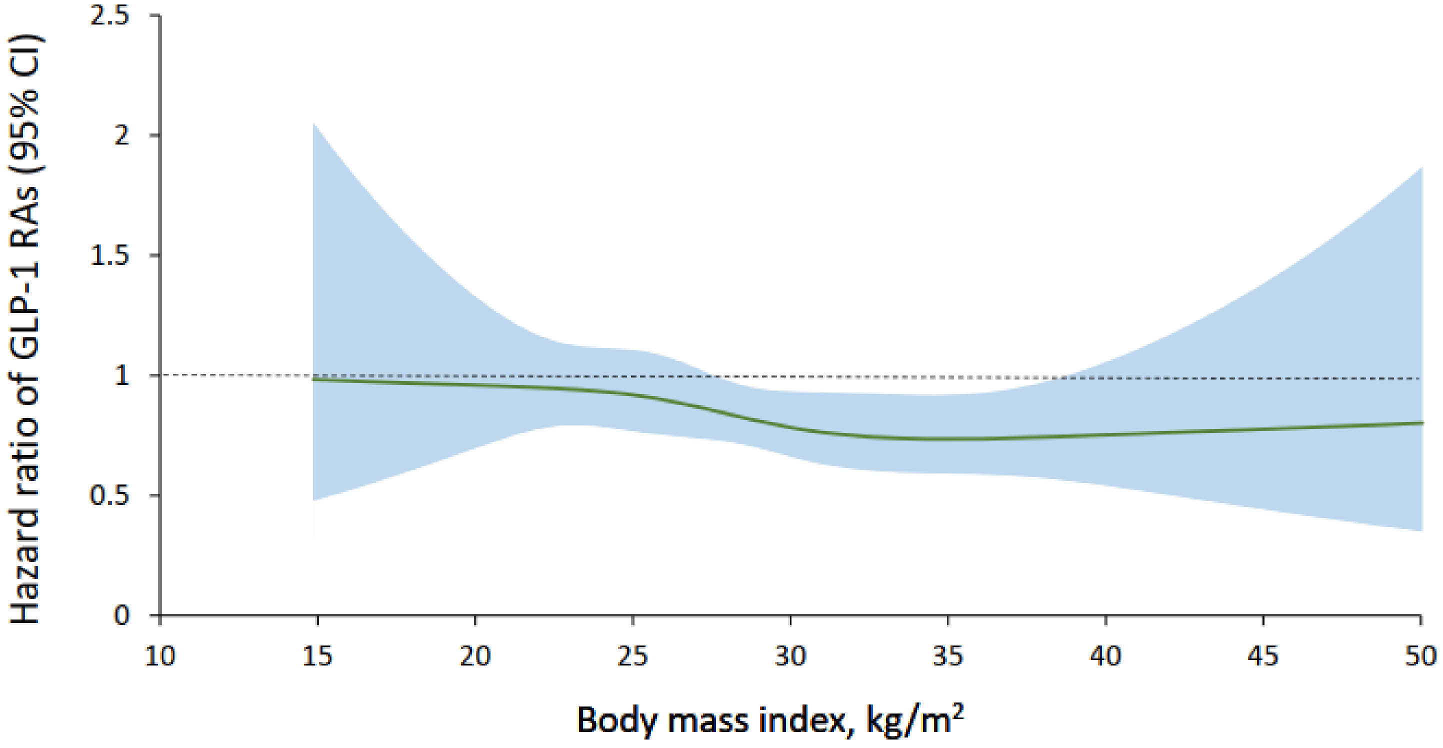

© 2025 Chen TH et al. *JAMA Network Open*.

C. Composite renal outcome

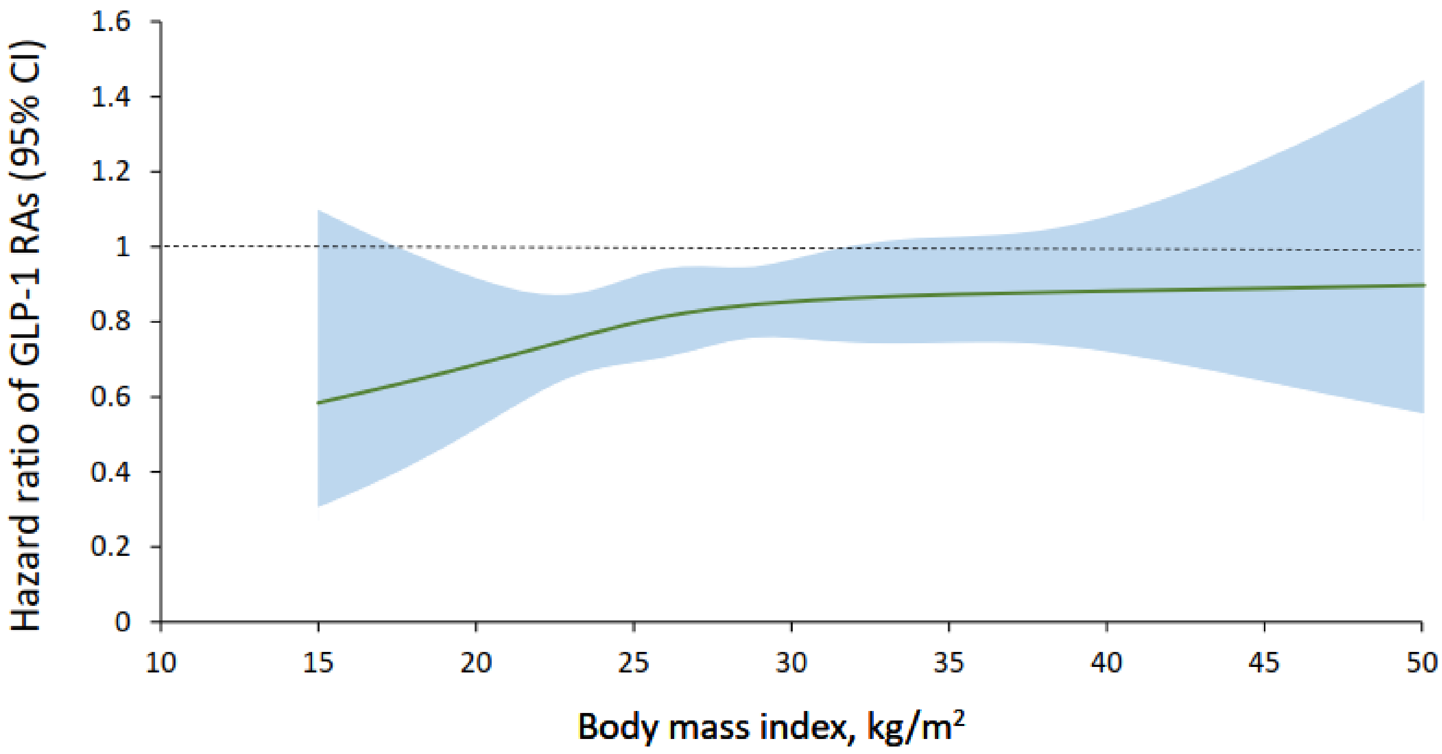

© 2025 Chen TH et al. *JAMA Network Open*.
