# Supplementary material for: GLP-1 RAs and Cardiovascular and Kidney Outcomes by Body Mass Index in Type 2 Diabetes
Source: JAMA Netw Open. 2025 Sep 8;8(9):e2530952. doi: 10.1001/jamanetworkopen.2025.30952 (PMC12418133; doi:10.1001/jamanetworkopen.2025.30952)
Supplement: Supplement 2. — Data Sharing Statement [file jamanetwopen-e2530952-s002.pdf]

Data Sharing Statement

Chen. GLP-1 RAs and Cardiovascular and Kidney Outcomes by Body Mass Index in Type 2 Diabetes. *JAMA Netw Open*. Published September 08, 2025.
doi:10.1001/jamanetworkopen.2025.30952

Data
Data available: No

Additional Information
Explanation for why data not available: The original contributions presented in this study are included in the article. The data presented in this study are available on request from the corresponding author. The data are not publicly available because the data in the CGRD can only be obtained and used inside Chang Gung Memorial Hospital.
